# A pathway for complete mineralization of the ambergris constituent (−)-Ambrox in *Sphingomonas* sp.

**DOI:** 10.1128/aem.00876-26

**Published:** 2026-08-05

**Authors:** Tina Haupt, Felix Flachsmann, Gerhard Brunner, Yumiko Weiner-Sekiya, Lucas Hortencio, Cyrille Jarin, Patrick Robe, Alessio Fonzo, Francis Voirol, Eric Eichhorn, Andreas Natsch

**Affiliations:** 1 Fragrances S&T, Ingredients Research, Givaudan Schweiz AG, Kemptthal, Switzerland; 2 Active Beauty, Givaudan France SA, Ramonville Saint Agne, France; Universidad de los Andes, Bogotá, Colombia

**Keywords:** biodegradation, complex pathways, polycyclic compound, terpene, *Sphingomonas *sp.

## Abstract

(−)-Ambrox, the smelling principle in ambergris of sperm whales, is a saturated polycyclic terpenoid ether with multiple tertiary and quaternary centers. Despite these structural features, it is readily biodegradable and thus represents an interesting case to understand the degradation pathway of a complex, saturated polycyclic terpene. We isolated two *Sphingomonas* spp. strains from activated sewage sludge capable of rapidly mineralizing (−)-Ambrox. The structures of seven isolated metabolites were determined by 2D-NMR, and further metabolite structures were proposed based on their exact masses. The use of two ^13^C-labeled substrates allowed completing the hypothesis for the biodegradation pathway. Biodegradation starts by oxidation of the α-C(4)-methyl group, followed by hydroxylation of the adjacent C(5)-angular CH-group, which allows opening of ring A by a unique retro-aldol process. β-Oxidation leads to a C11 metabolite, which is further degraded to a C6 δ-lactone. This metabolic fate is conserved among strains. The structure-biodegradation relationship indicates that only natural (−)-Ambrox and related labdanoids were mineralized, but not enantiomers and epimers. For both strains, the complete genome was sequenced; for one strain, a fosmid library was prepared, and the gene for the first oxidation step was cloned. The activity for the following step was located on a fosmid in a non-adjacent genomic region, indicating that this pathway is dispersed over the genome. This detailed study provides unique insights into the complex biodegradation pathway of the poorly studied class of saturated polycyclic terpenoids.

**IMPORTANCE:**

For complex, polycyclic natural substrates such as sesquiterpenes and diterpenes, there is little knowledge on biodegradation pathways, except for the elucidated steroid degradation pathways. This study presents key steps in the biodegradation pathway for (−)-Ambrox, a natural constituent of the sperm whale secretion ambergris, which was found to be readily biodegradable, despite a polycyclic ring system with multiple tertiary and quaternary carbon centers. This may be one of the first detailed studies on the biodegradation pathway of a polycyclic natural sesquiterpenoid.

## INTRODUCTION

**Fig 1:**
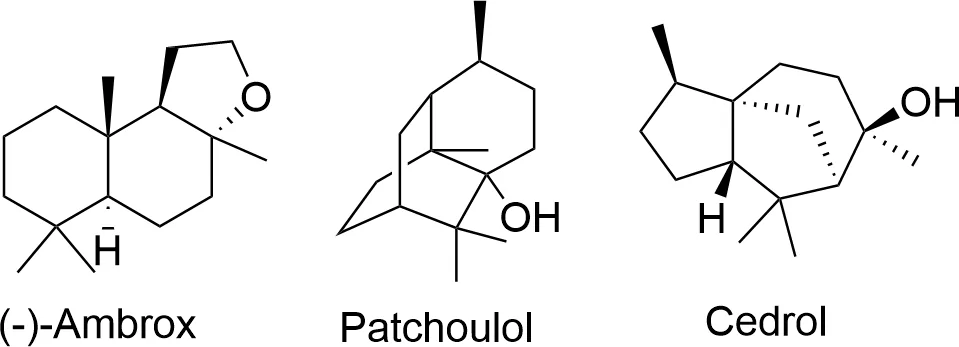
The structure of (−)-Ambrox and other polycyclic biodegradable sesquiterpenes for which no degradation pathway is known.

To develop novel biodegradable compounds for consumer products that are safe by design, understanding biodegradation pathways and reactions is key. Over the last few decades, research into biodegradation pathways has focused on (poly)aromatic compounds (1), (poly)halogenated compounds (2), nitrogen-containing pesticides, and drugs (3). The degradation pathways of complex saturated natural carbon skeletons have received little attention. In the structural class of terpenoids, a degradation pathway for the abundant monoterpene pinene has been proposed (4, 5), and camphor degradation by Baeyer-Villiger monooxygenases has been well studied (6). Most polycyclic sesquiterpenes, such as cedrol, cadinene, and caryophyllene, were shown to be biodegradable under screening conditions (7). Yet, for none of these compounds is the degradation pathway known, although for cedrol, multiple hydroxylation at different sites by different microorganisms has been reported (8–10). This lack of mechanistic understanding may explain the poor performance of predictive *in silico* biodegradation models for polycyclic saturated carbon skeletons (7). Several of these sesquiterpenes have multiple tertiary and quaternary carbon centers, structural features that typically hamper biodegradation. Some biodegradable sesquiterpenes, such as patchoulol and cedrol (Fig. 1), also lack double bonds as possible points of initial microbial attack. The most detailed knowledge has been gathered regarding the degradation of steroids, cholesterol, and cholic acid, which are used as nutrients by many microorganisms (11).

An interesting compound is (−)-Ambrox (Fig. 1), a rare and unique nature-identical fragrant compound and the main odor vector of ambergris from sperm whales (12), which is also found as a constituent in labdanum oil obtained from *Cistus ladanifer* (13, 14). It is a fully saturated tricyclic terpenoid ether with two tertiary and three quaternary centers. The presence of two quaternary carbons on both carbacyclic six-membered rings does not allow early aromatization followed by aromatic ring cleavage, which has been shown to be a key step in the microbial degradation pathways of steroids, such as estrogen (15) and testosterone (11), or abietane diterpenoids, such as the highly abundant abietic acid (16). The hindered tetrahydrofuran ring constitutes another structural element with a poor biodegradation precedent. Thus, it is expected that (−)-Ambrox would not undergo facile biodegradation; however, it was found to be readily biodegradable in Organisation for Economic Co-operation and Development (OECD) screening tests (17). Whereas hydroxylation at three different positions of (−)-Ambrox by different fungal isolates has been described (18–20), there are no reported indications for a pathway for (−)-Ambrox mineralization.

To better document the microbial degradation of such complex terpenoids, we prepared enrichment cultures by incubation of activated sludge with (−)-Ambrox. Two *Sphingomonas* sp. strains isolated from these cultures are capable of mineralizing the substrate. We studied the degradation pathway in detail by purifying the intermediary metabolites and determining their structures by 2D-NMR. Selective ^13^C-isotope labeling of the substrate at two methyl groups further helped to postulate the degradation pathway and the link between intermediates. We further synthesized or purchased 19 derivatives of (−)-Ambrox and related natural compounds and assessed their mineralization to generate a detailed structure-biodegradation relationship for the isolated strain. Natural substrates closely related to (−)-Ambrox, such as drimanol, sclareol, and manool, were mineralized by the same isolate. Based on full-genome sequencing and a fosmid library, the gene for the first oxidation step was isolated from one strain, and it was shown that the initial oxidation reactions in this complex pathway are performed by genes which are not organized in an operon. This study presents a novel degradation pathway for complex natural substrates and shows that a complete degradation pathway is present in single bacterial isolates.

## MATERIALS AND METHODS

### Collection of sewage sludge and enrichment for (−)-Ambrox degrading strains

Fresh activated sludge from a biological wastewater treatment plant treating predominantly domestic sewage (Bois-de-Bay, Satigny, Switzerland) was washed three times in OECD mineral medium (MM) amended with 1 mL/L trace element solution (Sigma-Aldrich), adjusted to a final density of 30 mg/L dry weight of organic matter, kept aerobic, and used on the same day as indicated in OECD TG 301F (21). (−)-Ambrox was tested at a concentration of 30 mg/L for 28 days under standard 301F conditions. The enriched sludge at the end of this ready biodegradation test was then diluted 25-fold in fresh MM and amended with 100 mg/L (−)-Ambrox. After 14 days, the sludge was harvested and incubated for another 14 days with fresh substrate in MM.

### Isolation of (−)-Ambrox degrading bacterial strains, verification of primary degradation, and mineralization by isolated strains

The enriched sludge was plated on 1/10 strength tryptic soy agar; the isolated colonies were purified, and the taxonomic relationship was determined by 16S rDNA sequencing. Isolated strains were grown in full-strength tryptic soy broth at 22.5°C and 150 rpm shaking for 4–5 days. Strains were washed, adjusted to OD_600_ = 1, incubated with 20 mg/L (−)-Ambrox in 1 mL MM for up to 7 days, extracted with 1 mL methyl-*tert*-butyl ether, and analyzed by gas chromatography for residual (−)-Ambrox to determine primary degradation. To confirm mineralization of (−)-Ambrox and related substrates, strains were incubated at a final OD_600_ = 0.005 in oxygen-saturated MM and incubated in completely filled, closed 21 mL glass vials at approximately 3 mg/L substrate concentration, whereby the exact substrate concentration was calculated so that full mineralization would lead to complete consumption of dissolved oxygen. The oxygen concentration during the biodegradation reaction was measured online using a SensorDish reader (PreSens, Regensburg, Germany) with an oxygen sensor spot fixed to the bottom of each vial.

### Preparation of resting cell culture, isolation of metabolites, and high-performance liquid chromatography high-resolution mass spectrometry analysis

To generate and isolate metabolites, the newly isolated strain Ambrox-5 was grown in full-strength tryptic soy broth, harvested from late exponential phase, and incubated at a final OD_600_ = 1 in 200 mL of MM with 100 mg/L (−)-Ambrox for up to 48 h. Samples were frozen at −80°C, thawed, and sonicated in 30 mL aliquots with an ultrasonic tip with a Branson sonicator (SFX 550) three times for 1 min (duty cycle 30%) with intermediate cooling, centrifuged at 4,000 rpm for 10 min, and the supernatant was loaded onto an OASIS HLB cartridge with 1 g sorbent (Waters 186000117 column). The cartridge was washed with 10 mL 5% methanol and eluted with 10 mL methanol. The eluate was then concentrated to 0.5 mL under an N_2_ stream. Prior to metabolite isolation, acetonitrile (0.5 mL) was added to the sample. Aliquots of 7 µL of this sample were injected on a Dionex UltiMate 3000 HPLC system coupled to a DAD-3000 Diode Array Detector and an AFC-3000 Automated Fraction Collector. For liquid chromatography, a 4.6 mm × 150 mm Waters XBridge BEH C18 column with a pore size of 130 Å and a particle size of 2.5 µm was used. The flow rate was 1.0 mL/min. Eluent A consisted of H_2_O:ACN 95:5 + 0.1% formic acid (vol/vol), and eluent B consisted of ACN:H_2_O 95:5 + 0.1% formic acid (vol/vol). A linear gradient was run from 100% A (held for 3 min) to 100% B within 17 min (held for 3 min), back to 100% A (held for 5 min). The column temperature was set to 30°C, and detection wavelength was set to 257 nm with a bandwidth of 5 nm and a reference bandwidth of 100 nm. Fraction collection was time-triggered, and fractions were collected in 60 mL glass tubes over a total of 92 injections. Prior to high-performance liquid chromatography high-resolution mass spectrometry (HPLC-HRMS) measurements, excess solvent in the fractions was removed using a rotary evaporator Büchi R-200, equipped with a V-500 vacuum system. Fractions chosen for 2D-NMR measurements were dried with a freeze dryer (Christ Alpha 2-4 LD plus) and then re-dissolved in the deuterated solvent of choice.

The complete eluate from the solid-phase extraction step and the individual fractions eluted from the preparative HPLC were analyzed with high-resolution HPLC-HRMS (LC-HRMS) on a Dionex UltiMate 3000 RS HPLC system coupled to a Q Exactive Orbitrap mass spectrometer (Thermo Scientific, Reinach, Switzerland) with electrospray ionization in positive and negative ionization modes. For liquid chromatography separation, a Waters XBridge C18 column with a pore size of 130 Å, dimensions 2.1 mm × 50 mm, and particle size of 2.5 µm, with a 2.1 mm × 5 mm pre-column with particle size of 2.5 µm of the same material, was used. The flow rate was 0.2 mL/min. Eluent A consisted of water containing 5% acetonitrile and 0.1% formic acid, while eluent B consisted of acetonitrile containing 5% water and 0.1% formic acid. A linear gradient was run from 10% eluent B (held for 1 min) to 70% eluent B in 6 min, followed by a linear increase to 100% eluent B in 1 min (held for 1.5 min), back to 10% eluent B followed by 1.5 min equilibration time. The injection volume for each sample was 10 µL. The mass resolution of the HR-MS spectra was set to 70,000. The mass accuracy was <5 ppm. Data-dependent high-resolution product-ion spectra (HR-MS/MS) were recorded at a resolution of 17,500. Ion source parameters adjusted were as follows: sheath gas flow (35 arbitrary units), auxiliary gas flow (10 arbitrary units), sweep gas flow (1 arbitrary unit), capillary temperature (300°C), and source voltage (4.5 kV in positive mode and 3.5 kV in negative mode). Fragmentation was obtained from dissociation in an octopole collision cell using higher-energy collision dissociation settings at a normalized collision energy of 35 (arbitrary units). The mass scan range was set from 50 to 750 *m*/*z*.

### Synthesis of substrates and reference compounds

(−)-Ambrox was obtained from Givaudan (Kemptthal, Switzerland). The synthesis and isolation of a library of stereoisomers of (−)-Ambrox have recently been described (22). Further substrates were either commercially available or synthesized according to literature procedures.

### (Bio)synthesis of ^13^C-labeled substrates

**Fig 2:**
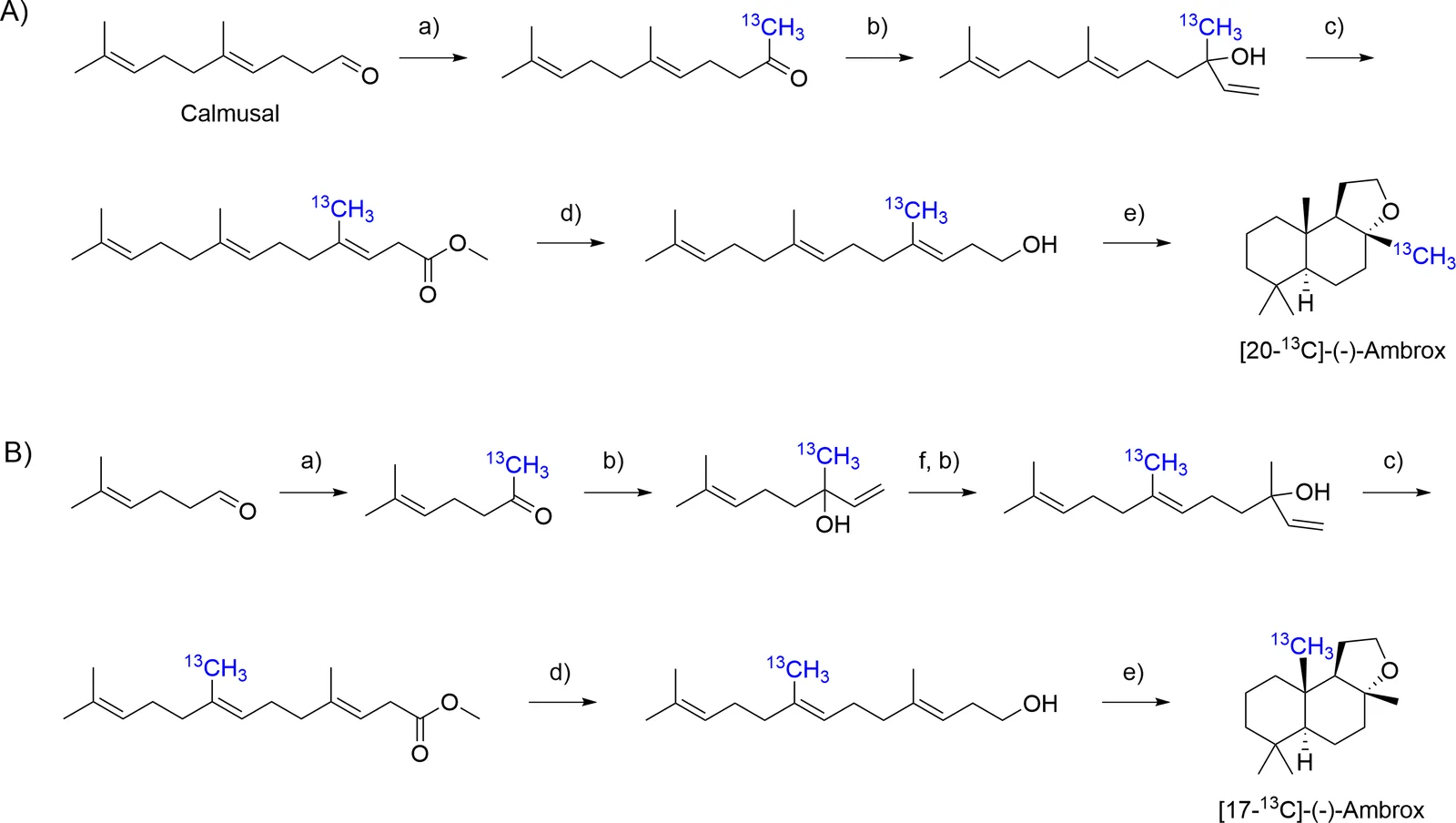
Synthesis of (−)-Ambrox with ^13^C-labels at C(20) (**A**) and C(17) (**B**). a) ^13^CH_3_MgI and PCC; b) vinyl-MgBr; c) (i) methyl chloroformate, NaH and (ii) CO (30 bar), cat. Pd(OAC)_2_/PPh_3_, Na-propionate, EtOH; d) RedAl; e) SHC (Eichhorn et al. [23], #13256); f) methyl isopropenyl ether, Δ (Saucy-Marbet reaction).

Homofarnesol samples labeled with either ^13^CH_3_(4) or ^13^CH_3_(8) were prepared according to the scheme below (Fig. 2). They were subjected to squalene-hopene-cyclase (SHC)-mediated polycyclization, as described previously (22, 23), to yield [20-^13^C] and [17-^13^C]-(−)-Ambrox. Details of the synthesis and analytical data are presented in the Supplemental material.

### 2D-NMR for structure elucidation

All NMRs were recorded using a Bruker Avance III 600 MHz NMR instrument equipped with a 1.7 mm TCI-microprobe. The isolated metabolites were dissolved in 35 μL C6D6 with the highest commercially available deuterium enrichment of 99.96% D, except for metabolite M11, which was dissolved in DMSO-D6, 99.95% D. To start, a ^1^H-NMR experiment allowed the estimation of the sample amount, which served to set the number of scans in the subsequent 2D-NMR gradient-enhanced experiments in a suitable manner. As a second step, the multiplicity-edited HSQC, HMBC, and COSY-DQF led to the flat structure elucidation of the metabolites. Finally, the relative configurations were attributed to the NOESY experiment.

### Bacterial genomic DNA purification

Frozen bacterial pellets of both strains Ambrox-5 and Ambrox 29-2 were gently thawed on ice and manually resuspended in 900 µL of lysis buffer I (10 mM Tris-HCl, 10 mM EDTA, 27.5 mg/mL lysozyme, 2.9 mg/mL achromopeptidase, 250 U/mL mutanolysin, and 400 U/mL lysostaphin). The suspension was incubated for 22 h at 37°C. Subsequently, 40 µL of RNase A (1 mg/mL) was added, followed by incubation for 30 min at 37°C. Lysis buffer II (900 µL; 10 mM Tris-HCl, 10 mM EDTA, 4 mg/mL proteinase K, 2% lauryl sarcosyl) was then added, and the mixture was incubated at 56°C for 24 h.

Proteins were removed, and DNA was purified using 5PRIME Phase Lock Gel Heavy tubes (Quantabio, 2302830), following the manufacturer’s instructions, with phenol:chloroform:isoamyl alcohol (25:24:1) as the organic phase. The residual phenol was removed by adding one volume of chloroform to the aqueous phase. Nucleic acids were precipitated using sodium acetate and ethanol without centrifugation; large DNA fragments, which formed a jelly-like mass (“medusa”), were gently collected using a sterile pipette tip. The fragments were washed twice with 1 mL of cold 70% ethanol, air-dried for 2 h at room temperature, and resuspended in 300 µL of 10 mM Tris-HCl.

### Sequencing and data analysis

The complete genomes were sequenced using both short- and long-read approaches: Illumina MiSeq for short reads and Oxford Nanopore GridION for long reads. MiSeq libraries were prepared using the TruSeq DNA Nano Low Throughput Library Prep Kit (Illumina, 20015964), and GridION libraries were prepared using the Ligation Sequencing Kit (ONT, SQK-LSK109), according to the manufacturer’s protocols.

Genome assembly was performed using a hybrid approach combining Illumina and Oxford Nanopore reads with Unicycler (v0.4.9b [24]) executed in the “conservative” mode. Unicycler utilized internal modules, including SPAdes (v3.13.0) for *de novo* assembly, Racon (v1.4.19) for long-read polishing, and Pilon (v1.22) for final polishing using short reads. Functional annotation of the assembled genomes was performed automatically using Prokka (v1.13 [25]).

### Fosmid library preparation

The genomic DNA of strain Ambrox 29-2 was cloned into fosmids using the pCC1FOS Fosmid Library Production Kit (Lucigen, CCFOS10), as recommended by the manufacturer. With this approach, an average insert size of 40 kb is obtained. Recombinant EPI300T1R *Escherichia coli* were manually transferred to four 96-well microtiter plates containing freezing medium (Luria-Bertani [LB], 8% glycerol complemented with 12.5 µg/mL chloramphenicol). After 22 h of growth at 37°C without any agitation, the plates were stored at −80°C. The library contained 384 clones and with a genome size for Ambrox 29-2 of 3,616,569 bp, a theoretical coverage of 4.2-fold was obtained, and for each genomic site, the probability to be covered is at 99%.

### Metabolism by fosmid clones

The fosmid clones (*n* = 384) in the host strain *E. coli* EPI 300 T1R were grown in 300 µL LB induction medium containing 12.5 µg/mL chloramphenicol and 10 mM arabinose in deep well plates incubated at a slight angle at 37°C and with 250 rpm shaking overnight. Samples (150 µL) from eight wells of a column were pooled into a 1.5 mL Eppendorf tube, followed by a centrifugation step at 14,400 rpm for 10 min at RT. The pellet was resuspended in 1 mL MM and transferred to 1.8 mL glass vials coated with substrate to achieve a final substrate concentration of 30 mg/L. The pools were incubated at 30°C for 4 days and 600 U/min, lysed with 75 µL lysozyme (10 mg/mL in 10 mM Tris-HCl buffer, pH 8.0) for 30 min at 30°C and 150 rpm, followed by centrifugation at 3,000 rpm for 10 min. The supernatant was purified on an OASIS HLB µElution plate 30 µm cartridge (Waters 186001828BA plate) and analyzed by HPLC-HRMS as described above. Single fosmid clones of active pools were evaluated in the same manner. For the DNA purification of the target fosmids, an overnight culture in LB supplemented with 12.5 µg/mL chloramphenicol followed by 5 h induction with CopyControl induction solution (Lucigen) was prepared. DNA purification of 25 mL induced overnight culture was performed using PureLink HiPure Plasmid DNA Purification Kits (K2100-02). Sequencing of active fosmids with pCC1 primers was performed using Microsynth (Balgach, Switzerland).

### Expression of target genes

Hypothetical candidate genes present in the active fosmids were synthesized, cloned into the pET-24a(+) vector, and transformed into *E. coli* strain BL21(DE3). Recombinant strains were selected on LB plates supplemented with 50 µg/mL kanamycin and grown in 20 mL LB broth supplemented with 50 µg/mL kanamycin, followed by 24 h of induction in auto-inducing TB medium. Cells were harvested by centrifugation at 5,000 rpm for 15 min at RT, resuspended in MM to a final OD_600_ = 1 or 10, and incubated with the substrates for 24 h to 4 days. The samples were analyzed for metabolites using HPLC-HRMS, as described above.

## RESULTS

### Isolation and characterization of (−)-Ambrox degrading strains

**Fig 3:**
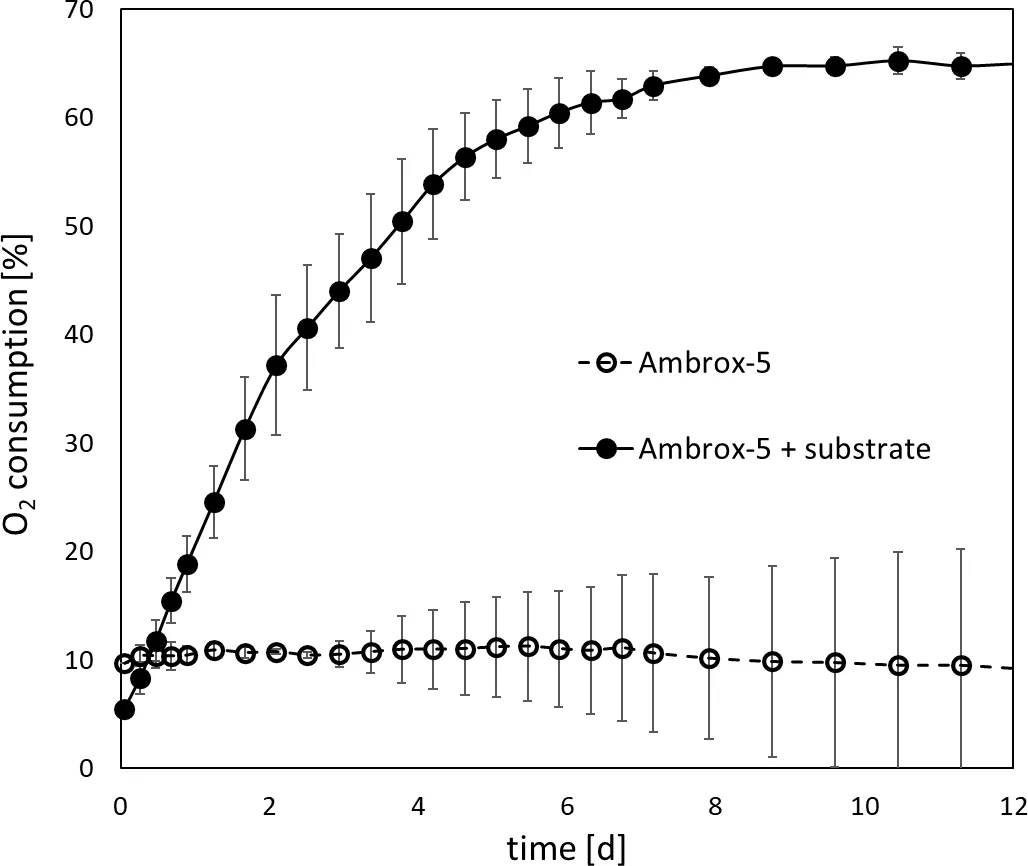
Mineralization of (−)-Ambrox by *Sphingomonas* Ambrox-5. Representative experiment of *n* = 7 independent studies. Ambrox-5 at an inoculum of OD_600_ = 0.005 was incubated with 3 mg/L of (−)-Ambrox in an oxygen-saturated buffer, and residual dissolved oxygen was monitored by an optical sensor.

(−)-Ambrox is readily biodegradable under the conditions of the OECD 301F test (17). Thus, we conducted a 301F test with 30 mg/L of substrate and used the adapted bacterial community after 28 days to further enrich for (−)-Ambrox-degrading strains over 28 days. Single isolates of the adapted culture were collected, screened for primary degradation, and analyzed for their 16S rDNA sequence. Two isolates, nicknamed Ambrox-5 and Ambrox-29-2, showed complete primary degradation of (−)-Ambrox and were selected for further analysis. Based on the 16S gene, Ambrox-5 and Ambrox-29-2 are closely related to *Sphingomonas* sp. BA1, an isolate investigated for its tolerance to high concentrations of nickel ions (26). Full-genome sequencing of Ambrox-5 and Ambrox-29-2 (see below) confirmed that both strains belong to the genus *Sphingomonas* but that they are also significantly different, e.g., in terms of genome size. Ambrox-5 was further assessed for mineralization of (−)-Ambrox in a setup mimicking the condition of OECD test 301D, whereby the substrate was dissolved at a low concentration in an oxygen-saturated buffer. Ambrox-5 at an inoculum of OD_600_ = 0.005 led to 62.8% ± 5.3% (*n* = 7 independent experiments) of biological oxygen demand reaching a plateau after 8 days (Fig. 3), showing that this isolated strain on its own can mineralize (−)-Ambrox. Similar results were obtained for Ambrox-29-2 (data not shown).

### Isolation and structure elucidation of (−)-Ambrox metabolites

As shown in Table S1, strain Ambrox-5 did accumulate higher levels of intermediate metabolites when incubated with (−)-Ambrox compared to Ambrox-29-2 and hence was chosen for isolation of the metabolites. A resting cell culture biotransformation was thus run with Ambrox-5 cells at OD_600_ = 1 together with 100 mg/L (−)-Ambrox. Samples were taken over time, quenched, and extracted at different time points. Multiple metabolites were present, as observed by HPLC-HRMS analysis (for a representative analysis, see Fig. S1). The culture of Ambrox-5 harvested after 20 h, containing the highest amounts of metabolites, was sonicated, metabolites were extracted by solid-phase extraction, and subjected to preparative HPLC. Seven metabolites were detected in sufficient quantities for isolation. The structures of these metabolites were determined using 2D-NMR analysis (M1–M6 and M10/M11; Table 1 and Fig. S2–S16).

**TABLE 1:** Structure of metabolites elucidated by 2D-NMR in resting cell culture transformation of (−)-Ambrox with Ambrox-5

Metabolite	Structure	Rt (min)	Formula Exact mass (g/mol)	Observed ions in HPLC-HRMS (g/mol)	NMR data
**M1**	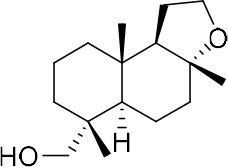	6.76	C_16_H_28_O_2_ 252.2089	253.2162 [M + H]^+^ 235.2056 [M − H_2_O + H]^+^	Fig. S2–S4
**M2**	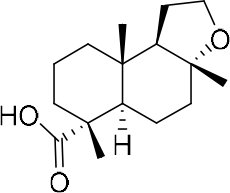	6.65	C_16_H_26_O_3_ 266.1882	267.1955 [M + H]^+^ 265.1809 [M − H]^−^	Fig. S5 and S6
**M3**	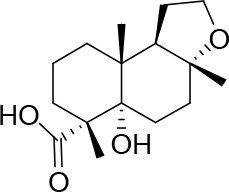	5.97	C_16_H_26_O_4_ 282.1831	283.1904 [M + H]^+^ 265.1798 [M − H_2_O + H]^+^ 305.1723 [M + Na]^+^ 281.1758 [M − H]^−^	Fig. S7–S9
**M4**	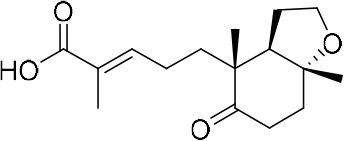	5.88	C_16_H_24_O_4_ 280.1675	281.1748 [M + H]^+^ 263.1642 [M − H_2_O + H]^+^ 303.1567 [M + Na]^+^ 279.1602 [M − H]^−^	Fig. S10 and S11
**M5**	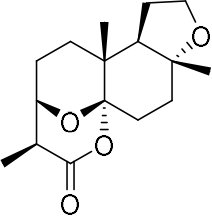	5.43^*a*^	C_16_H_26_O_5_^*a*^ 298.1780	299.1853 [M + H]^+^ 281.1747 [M − H_2_O + H]^+^ 321.1672 [M + Na]^+^ 297.1707 [M − H]^−^	Fig. S12 and S13
**M6**	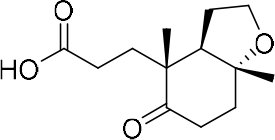	5.28	C_13_H_20_O_4_ 240.1362	241.1435 [M + H]^+^ 223.1329 [M − H_2_O + H]^+^ 263.1254 [M + Na]^+^ 239.1289 [M − H]^−^	Fig. S14 and S15
**M10**	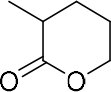	4.25	C_6_H_10_O_2_ 114.0681	115.0754 [M + H]^+^ 97.0648 [M − H_2_O + H]^+^ 137.0573 [M + Na]^+^	
**M11**	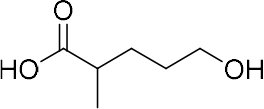	Upon isolation, M10 hydrolyzed and was analyzed by NMR as δ-hydroxy acid			Fig. S16

^*a*^ LC-HRMS detection in hydrolyzed form, that is, 3-hydroxy-acid after the hydration of M4. M5 can be present in different open and lactonic forms, and the NMR data were consistent with these three different forms, as shown in Fig. S12 and S13.

Based on the HSQC spectra (Fig. S2), a structure for metabolite **M1** was proposed with one of the four methyl groups replaced by a primary alcohol, resonating at 72.0 ppm in the ^13^C-NMR spectrum. Moreover, the HMBC experiments (Fig. S3) showed that this new functionality is linked to a methyl group by a 3-bond correlation, indicating that one methyl group, 18 or 19, of the *gem*-dimethyl of (−)-Ambrox had been oxidized. Therefore, only structural verification using HSQC, HMBC, COSY-DQF, and NOESY experiments was necessary to establish the structure of **M1**. Going one step further, metabolite **M3** was compared with **M1**. The newly discovered primary alcohol was no longer present. In contrast, HMBC (Fig. S8) revealed a carboxylic acid correlated to a methyl group, which showed that the primary alcohol was oxidized to a carboxylic acid. Additionally, the methine located at the ring junction of cycles A and B disappeared in favor of a tertiary alcohol, as suggested by HMBC. In this study, a structural hypothesis exhibiting these two modifications was verified for **M3** by applying the same four 2D-NMR experiments cited earlier (Fig. S7–S9). **M4** includes a double bond conjugated with the previously discovered carboxylic acid in **M3** and a ketone in the residual ring B, in addition to the intact ring C. Finally, the metabolite size decreases along the degradation pathway, leading to simpler molecules, such as δ-lactone **M10** or its hydrolyzed δ-hydroxy acid **M11**. When the same culture was sampled over 7 days, the early metabolites **M1** and **M2** appear early and disappear after 24 h, **M3** has a peak at 24 h while **M5**, **M6**, and **M11** accumulate later (Fig. S21), and this time course confirms the metabolic sequence.

Evidence for the presence of metabolites **M7–M9** (see Fig. 5) comes from HPLC-HRMS analysis, which provided exact masses matching the molecular formulae of these metabolites. These latter metabolites did not accumulate to the levels required for preparative isolation. However, they are typical products of β-oxidation, and hence, are biochemically plausible. An attempt was made to identify further metabolites from the degradation of **M6** by isolating **M6** by preparative HPLC, followed by incubation with Ambrox-5 cells and sampling after short incubation times (15 min to 24 h). The terminal metabolite **M11** started to accumulate already after 15 min, and no additional intermediate metabolites could be detected by HPLC-HRMS after SPE. This indicates that (i) **M11** is indeed a terminal degradation product of **M6** and (ii) the intermediate steps are fast (data not shown). In additional experiments, metabolite profiles were compared between two independent isolates, Ambrox-5 and Ambrox 29-2. This analysis showed that both strains produced **M1–M6**, followed by the terminal metabolite **M11**, indicating that the metabolic sequence of (−)-Ambrox is conserved between distinct members of this species (Table S1).

### Identifying metabolites from ^13^C-labeled substrates

**Fig 4:**
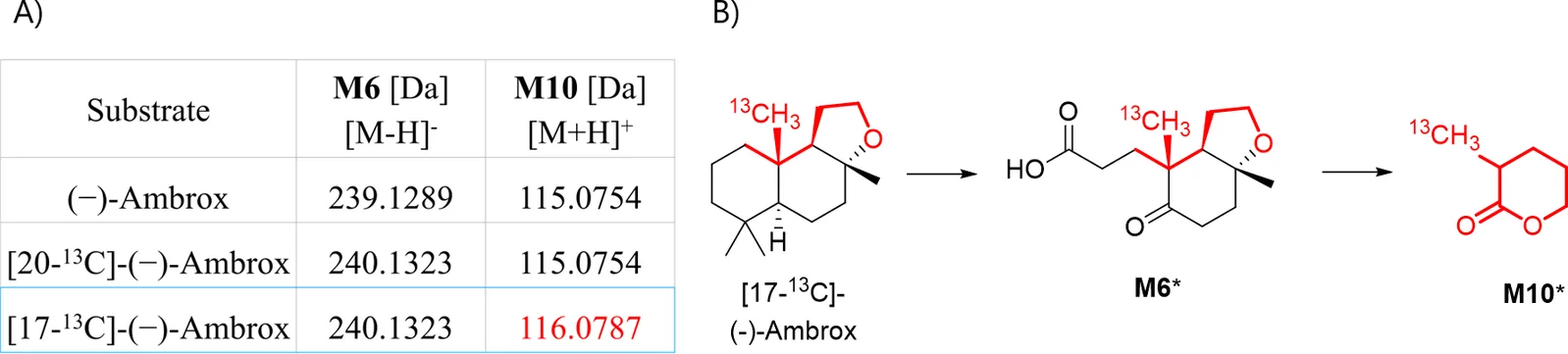
Degradation of ^13^C-labeled (−)-Ambrox by *Sphingomonas* sp. strain Ambrox-5. The three substrates were incubated (30 mg/L) in a resting cell culture biotransformation with cells at OD_600_ = 1 and metabolites analyzed after 4 h, 1 day, and 3 days with LC-HRMS. (**A**) Observed masses of metabolites M6 and M10. The corresponding masses were detected at all three time points. (**B**) Positioning of M10* subunit (red) in [17-^13^C]-(−)-Ambrox. See also Fig. S19 for the full metabolic sequence and Fig. S20 for quantitative data.

Because of the large gap in NMR-confirmed metabolites between **M6** and **M10/M11**, additional information was needed to formulate a complete degradation pathway for (−)-Ambrox. Specifically, we needed to determine whether the α-CH_3_ group in **M10/M11** originated from CH_3_(17) or CH_3_(20) in (−)-Ambrox. For the preparation of the required enantiopure ^13^C-labeled (−)-Ambrox substrates, the corresponding homofarnesol substrates (*E*,*Z* mixtures) were synthesized as shown in Fig. 2, followed by SHC-mediated polycyclization (23). The labeled substrates were incubated at a concentration of 30 mg/L in a resting cell culture biotransformation reaction with cells at OD_600_ = 1, and metabolites were analyzed after 4 h, 1 day, and 3 days by HPLC-HRMS. As shown in Fig. 4 and Fig. S19 and S20, the ^13^C label was retained in all early metabolites **M1–M6** for both labeled substrates. The ^13^C label was lost in **M10** for the C20-labeled substrate but was retained in **M10** for the C17-labeled substrate. This observation confirms (i) that the isolated metabolite **M10/M1**1 is indeed a late metabolite originating from the parent molecule and (ii) that, based on the retention of the labeled methyl group, **M10/M11** comes from the part of the parent highlighted in red in Fig. 4.

### A proposed pathway for (−)-Ambrox degradation

**Fig 5:**
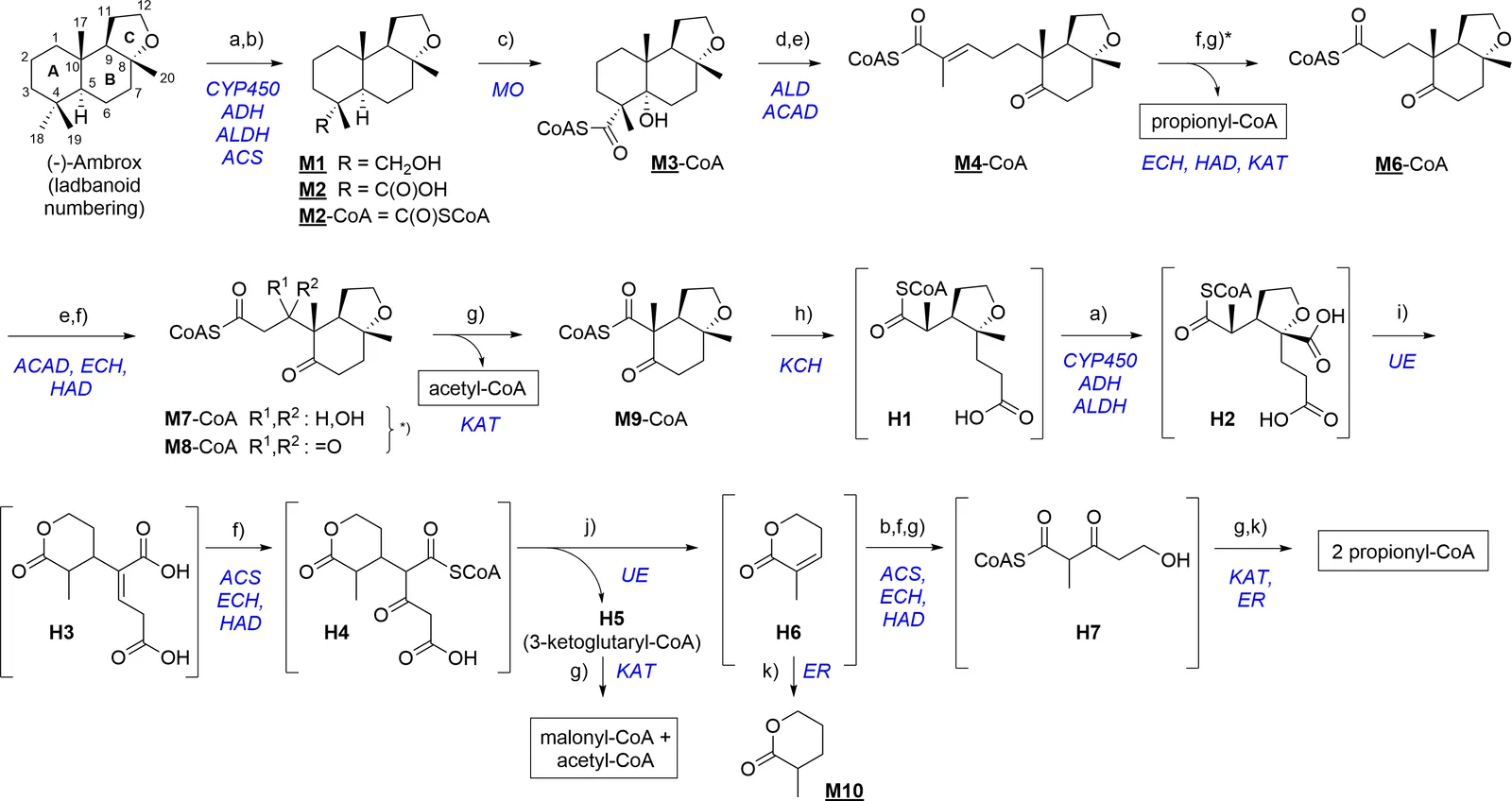
Proposed pathway for (−)-Ambrox degradation by *Sphingomonas* sp. strain Ambrox-5. Underlined metabolites **M1–6** and **M10** have been elucidated by NMR, whereas for **M7–9,** the corresponding exact masses have been detected. The hypothetical structures **H1–H7** were not observed. a) Hydroxylation of methyl group, stepwise oxidation to carboxylic acid, b) CoASH ligation, c) CH(5) hydroxylation, d) retro-aldol reaction, e) dehydrogenation, f) hydration, oxidation to ketone, g) thiolysis, h) retro-Dieckmann reaction, i) acid-catalyzed THF opening followed by spontaneous lactonization, j) retro-Michael reaction, k) enoate reduction. Abbreviations for postulated enzymes are as follows: CYP450: cytochrome P450 monooxygenase; ADH: alcohol dehydrogenase; ALDH: aldehyde dehydrogenase; ACS: acyl-CoA synthetase; MO: monooxygenase; ALD: aldolase; ACAD: acyl-CoA dehydrogenase; ECH: enoyl-CoA hydratase; HAD: 3-hydroxyacyl-CoA dehydrogenase; KAT: 3-ketoacyl-CoA thiolase; KCH: 2-ketocyclohexanecarboxyl-CoA hydrolase; UE: unknown enzyme or spontaneous; ER: enoate reductase. “*” indicates that the observed metabolite **M5** is a β-oxidation intermediate and was omitted for clarity.

Based on the isolated metabolites and the ^13^C-labeling experiment, the degradation pathway depicted in Fig. 5 was proposed. This hypothesis accounts for all elucidated intermediates, as well as the observed outcome with isotopically labeled substrates. The degradation starts with oxidation at the equatorial CH_3_(18) to form **M1** and **M2**. A key hydroxylation step at C(5), forming **M3,** prepares the stage for the cleavage of ring A by a retro-aldol reaction. The ring-opened product is subsequently desaturated to form **M4**. An alternative pathway involving additional hydroxylation of **M3** at C(3) followed by ring cleavage through Grob-type fragmentation would directly lead to **M4** under elimination of water. Degradation of **M4** by two cycles of β-oxidation, forming intermediates **M5–M8,** releases propionyl-CoA and acetyl-CoA under the formation of **M9**.

Ring B is proposed to be cleaved by a retro-Dieckmann condensation of **M9**-CoA (step h) to form **H1**, a transformation that has been reported on several occasions as part of the degradation pathways of steroids (e.g., formation of M10 in the degradation of estrogen [15] or cleavage of the steroid D ring in the common intermediate HIP by the enzyme enoyl-CoA hydratase 20 [EchA20] [27]). Stepwise oxidation of CH_3_(20) is proposed to produce **H2**, which undergoes opening of the THF ring (mediated by intramolecular protonation), followed by δ-lactone formation under the release of CoA (step i). The glutaconic acid side chain of **H3** is proposed to be activated as a CoA-ester, hydrated, and oxidized to **H4,** from which 3-ketoglutaryl-CoA is released via a retro-Michael reaction (step j). Finally, the unsaturated lactone **H6** is hydrolyzed and conjugated to CoASH, followed by β-oxidation to propionyl-CoA and 3-hydroxypropionic acid. The latter is then converted to propionyl-CoA via acryloyl-CoA. The fact that a small part of **H6** is reduced to slowly degrading **M10** is fortuitous for the elucidation of this biodegradation pathway. The pathway described herein is further supported by the fact that incubation of *Sphingomonas* sp. strain Ambrox-5 with isolated **M6** resulted in the formation of 3-(methyl)tetrahydro-2H-pyran-2-one (**M10**), thus confirming **M6** as a true intermediate in the degradation pathway to form **M10**.

In addition to hydride equivalents from the dehydrogenation steps, the proposed pathway yields three propionyl-CoA, two acetyl-CoA, and one malonyl-CoA from the degradation of one (−)-Ambrox molecule. If we assume conversion of propionyl-CoA to acetyl-CoA via pyruvate, which is a known bacterial metabolic pathway for propionic acid, the breakdown of one molecule of (−)-Ambrox yields six acetyl-CoA. Thus, the metabolic harvest of (−)-Ambrox is equivalent to that of three glucose molecules.

### High stereoselectivity: structure-biodegradation relationship for strain Ambrox-5

**Fig 6:**
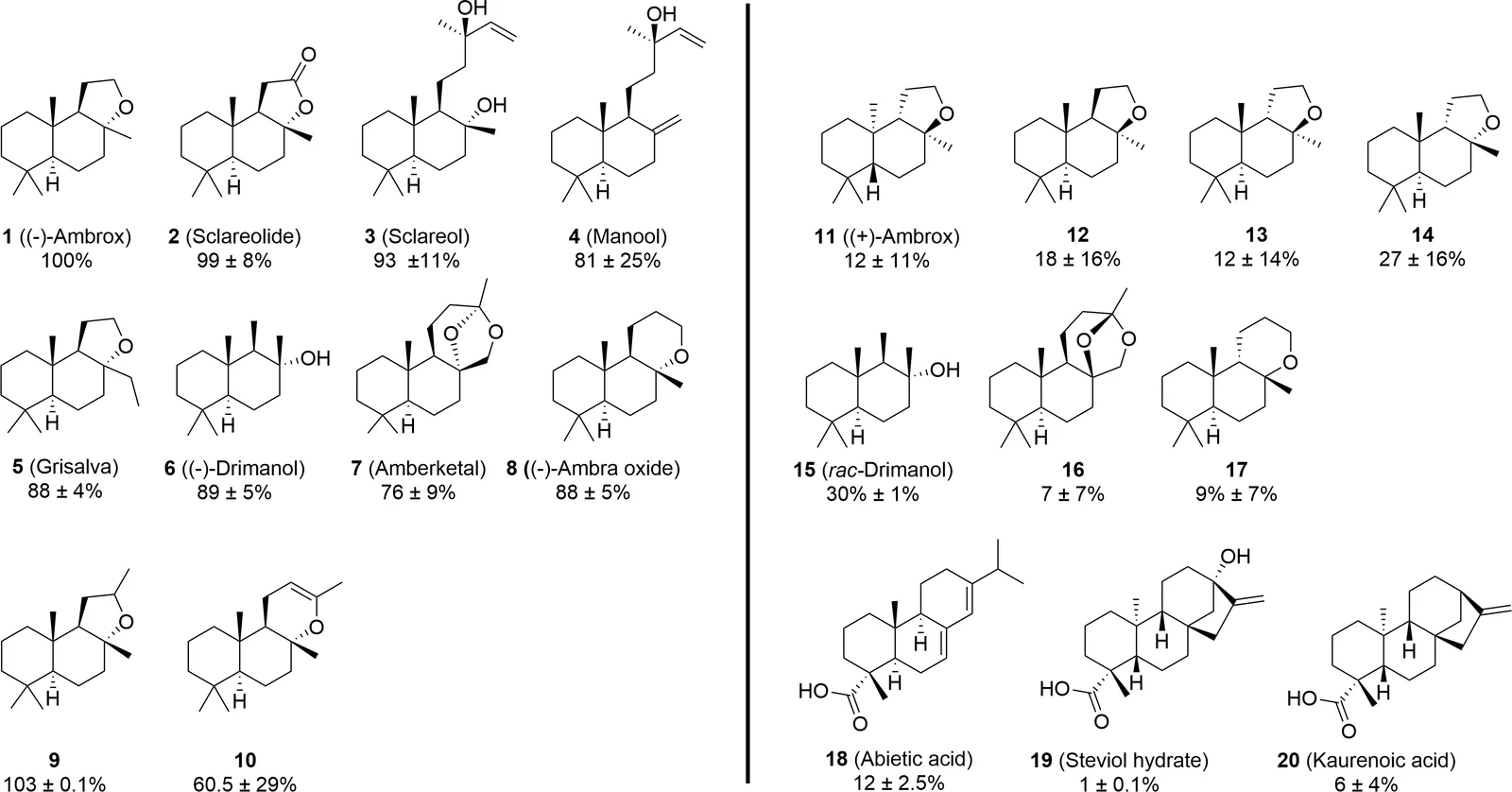
Substrate specificity for the mineralization ability of strain Ambrox-5 toward different natural and synthetic analogs related to (−)-Ambrox. Ambrox-5 at an inoculum of OD_600_ = 0.005 was incubated with the substrates in an oxygen-saturated buffer, and residual dissolved oxygen was monitored by an optical detector. Substrate concentration was adjusted so that the theoretical biological oxygen demand is equivalent to the maximal theoretical dissolved oxygen concentration. In each experiment, (−)-Ambrox was used as a positive control, and % mineralization is given with the value for (−)-Ambrox set to 100% in that given experiment. The absolute mineralization value for (−)-Ambrox is 62.8% ± 5.3%. Shown are the average and standard deviations from 2 to 3 independent experiments.

We further investigated the substrate specificity of the mineralization ability of strain Ambrox-5 using a wide range of natural and synthetic analogs of (−)-Ambrox. As summarized in Fig. 6, strain Ambrox-5 can efficiently mineralize several terpenoids that are closely related to (−)-Ambrox, such as sclareolide **2**, sclareol **3**, manool **4,** and drimanol **6**. HPLC-HRMS analysis of biotransformations run with strain Ambrox-5 and sclareol or manool revealed the exact masses of metabolites analogous to **M1–M6** of (−)-Ambrox (Table S2), which suggests that the same biodegradation pathway is used (Fig. 5), and that this pathway is of more general relevance for the degradation of natural compounds. On the other hand, the structurally related natural product abietic acid (**19**) is not significantly mineralized by strain Ambrox-5, despite the fact that it has the same absolute and relative configuration as (−)-Ambrox and its α-C(4)-methyl is already present in carboxylic acid form. For steviol hydrate and kaurenoic acid (**20–21**), the absence of mineralization may be attributed to their inverse absolute configuration compared to (−)-Ambrox (ent-kauranes). The synthetic Ambrox analogs **5** and **7–10** were degraded by strain Ambrox-5, which shows that the mineralization pathway tolerates modifications at the C-ring, as long as the absolute and relative configuration of the labdanoid skeleton is conserved. For (+)-Ambrox (**11**) or Ambrox stereoisomers **12–14**, no significant mineralization was observed. This high stereoselectivity for the natural configuration is further illustrated by comparing the different mineralizations of the pairs **7** vs **16** and **8** vs **17**, or the difference observed when testing pure drimanol **6** vs *rac*-drimanol **15**.

### Genome sequencing of strains Ambrox-5 and Ambrox-29-2

The genomes of both strains were sequenced using a hybrid assembly strategy to ensure high continuity. The genome of strain Ambrox-5 has a size of 4.83 Mb, distributed on a chromosome with 4.3 Mb and three plasmids, containing 4,515 open reading frames (ORFs) with 41% hypothetical proteins. The assembled genome had a GC content of 62.49%, and functional annotation identified 50 tRNAs and 6 rRNAs. Based on the full-genome sequence, this strain was most closely related to *Sphingomonas* sp. strain PAMC28499. The genome of Ambrox 29-2 has a size of 3.6 Mb, with a 3.1 Mb chromosome and two plasmids, containing 3,463 ORFs with 41% hypothetical proteins and 49 tRNAs. The genome sequence indicated *Sphingomonas indicum* was the closest relative. Completion of these closed genomes provides a definitive map of the accessory gene pool, which is essential for identifying the specific pathways involved in biodegradation. The annotation results and assembly metrics for strains Ambrox 29-2 and Ambrox-5 are listed in Table S3.

### Isolation of fosmid clones and target genes for the first steps of (−)-Ambrox degradation

It is remarkable that different, loosely related bacterial strains harbor a catabolic pathway forming the same key metabolites for complete mineralization of (−)-Ambrox (Table S1) and related natural substrates. We thus wondered whether this pathway is organized as a specific operon containing multiple enzymes and might therefore be conserved and inherited as a functional unit similar to the catabolic pathways of steroids (28) and other known biodegradation pathways. Therefore, we generated a fosmid library from one of these strains (Ambrox 29-2). Strain 29-2 had been chosen for fosmid library preparation before it was found that Ambrox-5 is actually more amenable to metabolite isolation, but since both strains produced the same metabolites, the further genetic work was done with this strain. A total of 384 fosmids covering the entire genome were grown, and pools from eight fosmid cultures were incubated with 30 mg/L (−)-Ambrox, and metabolites were analyzed by HPLC-HRMS. One pool was found to form metabolite **M1**, and a single fosmid clone [Plate 4-H5] from this pool was confirmed to generate **M1**. However, this fosmid clone did not generate any of the downstream metabolites, showing that it only contained the gene for the first step and not an operon for multiple steps. The candidate genes included two genes, CamC-1 and CamC-2 (protein sequences in supplemental material, page 25), related to camphor-5-monooxygenase (CamC). These candidate genes were expressed in *E. coli*. The *E. coli* cultures with the recombinant enzymes were then assessed for metabolism of (−)-Ambrox with HPLC-HRMS, and both genes related to CamC indeed formed M1 from (−)-Ambrox (Table 2), and hence have a redundant function. They are both located on the same fosmid, but interestingly have only very limited sequence homology to each other (172/426 [40%] identities and 232/426 [54%] positives), but both catalyze the selective oxidation of the methyl group in the *gem-*dimethyl group.

**TABLE 2:** Formation of M1 from (−)-Ambrox by the identified fosmid clone and the active genes CamC-1 and CamC-2

Clone	Condition	Peak area M1^*a*^
*E. coli* EPI 300 T1R no insert	OD = 1, 96 h	b.d.
*E. coli* EPI 300 T1R/fosmid [Plate 4-H5]	OD = 1, 96 h	1.97 × 10^7^
PET-24 a/BL21(DE3) no insert	OD = 1, 24 h	b.d.
PET-24 a/BL21(DE3)-CamC-1	OD = 1, 24 h	8.69 ± 1.05 × 10^7^
PET-24 a/BL21(DE3)-CamC-2	OD = 1, 24 h	3.81 ± 0.67 × 10^7^
PET-24 a/BL21(DE3) no insert	OD = 10, 24 h	b.d.
PET-24 a/BL21(DE3)-CamC-1	OD = 10, 24 h	1.42 ± 0.34 × 10^9^
PET-24 a/BL21(DE3)-CamC-2	OD = 10, 24 h	1.08 ± 0.40 × 10^9^

^*a*^ Shown is the peak area in HPLC-HRMS analysis for metabolite M1 formed from (−)-Ambrox in cultures of recombinant *E. coli* with the corresponding fosmids/plasmids. A fosmid pool and single clone (Plate 4-H5) after deconvolution transformed (−)-Ambrox to M1, but did not further transform it to M3. Both CamC-1 and CamC-2 on the fosmid [Plate 4-H5] conduct the same reaction. b.d., below detection.

A key committed step in the subsequent metabolic route is the specific (CH5)-hydroxylation to form **M3**. To find the responsible enzyme, we purified **M2** from a resting cell culture transformation using preparative HPLC and screened the fosmid library with this substrate. This led to the isolation of two fosmid clones ([Plate 3-C12] and [Plate 2-H2]) that could form **M3** from **M2** (Table S3). These fosmids were located in the same chromosomal region and are largely overlapping, containing 15 open reading frames in the forward direction. *E. coli* BL21 harboring either of these fosmids did not form further downstream metabolites (Table S3), indicating that this key oxidation step is also not part of an operon that catalyzes multiple steps. The overlapping parts of these two fosmids contained no obvious candidate genes, except ORF JDHFLCAE_00188, which encodes a putative oxidoreductase. However, *E. coli* BL21 cells expressing this gene did not convert **M2** into **M3**. In a further attempt to pinpoint the activity of these two fosmids to a single enzyme, the fosmid was distributed into seven subregions, each containing 1–3 ORFs. These were synthesized, introduced into pET-24a (+), and transformed into *E. coli* strain BL21(DE3). However, none of these subregions was active on their own when evaluated for the transformation of **M2** to **M3**, indicating that essential regulatory regions or co-factors for expression were lost in this subcloning step. Alternatively, **M2** may first need to be transformed to a CoA-ester to be further oxidized, as shown in the case of the biosynthesis of platensimycin (29), and indeed these two fosmids do contain a medium-chain fatty acid-CoA ligase (fadK), which might be required. However, given that this pathway is not organized as an operon, it will remain the subject of further studies to pinpoint all required genes.

## DISCUSSION

(−)-Ambrox is not an abundant constituent in nature, and to the best of our knowledge, it has only been described as a constituent of ambergris from sperm whales (12) and in the oil obtained from the resin of *Cistus ladanifer* (13, 14). Given its rare natural occurrence, it is rather surprising that widely occurring microbes in sewage treatment plants contain a complete degradation pathway for this substrate. (−)-Ambrox is now widely obtained from industrial production and used in perfumery (30), but due to its high price and high odor impact, it is used at low concentrations, and the overall input level in the influent in sewage treatment plants is very low (it may be estimated to be clearly below 1 ppb); hence, it is unlikely that this degradation pathway has evolved as a response to the availability of this substrate from commercial human activity. Manool is a component widely present in different tree species (31), and therefore the ecological significance and evolutionary origin of the observed degradation capacity may actually originate from the ability of strain Ambrox-5 and related strains to mineralize more widely available substrates such as manool.

The starting point of the pathway, the stepwise oxidation of a methyl group to the carboxylic acid, is a common metabolic step both in microorganisms and in hepatic metabolism in eukaryotes. The formation of **M3** by hydroxylation of the sterically crowded angular CH(5) is however surprising. This oxidation is a key prerequisite for the cleavage of the decalin substructure via a retro-aldol reaction. We have identified this metabolic sequence purely from NMR data of the isolated metabolites and only afterward discovered that there is a precedent: The biosynthesis of the antibiotic platensimycin starts with the formation of an *ent*-kaurene diterpenoid (32) with a carboxylic acid at the *gem*-dimethyl group, which is then hydroxylated at the same position of the decalin substructure by a specific oxidase (29) to set the stage for the subsequent A-ring cleavage. Nature thus uses the same metabolic reactions for catabolic degradation of natural terpenes and biosynthesis of a functional secondary metabolite. It is interesting to note that in contrast to the (−)-Ambrox degradation pathway by the herein described *Sphingomonas* spp., the enzymes involved in the complete biosynthesis of platensimycin are organized in a large operon. Since no intermediate metabolites between **M9** and **M10** accumulated and additional metabolites could also not be generated by using **M6** as substrate, the subsequent sequence until **M10** is currently fully hypothetical, although partly based on related reactions known from other pathways. Further research would be required to fully elucidate those steps.

The apparent theoretical oxygen consumption reaches 63%. This incomplete consumption under such conditions may at least partly be attributed to biomass build-up. Because (i) we find **M10** in all experiments, and (ii) since it is a terminal product from the intermediate M6 based on the labeling experiment and (iii) no alternative intermediate metabolites accumulated, we conclude that the pathway leads to full mineralization of the parent, and there is no branching into a non-productive terminal metabolite, with the exception of maybe build-up of small amounts of **M10**. The most closely related class of substrates which had been investigated in detail for biodegradation are steroid hormones (11, 28), cholic acid, and cholesterol. In contrast to the (−)-Ambrox studied herein, the absence of C(4) methyl groups in steroids enables degradation via aromatization of the A-ring and subsequent degradation of the aromatic ring via catechol intermediates (15). A well-studied step in the degradation of testosterone or cholesterol is the hydroxylation of C(9) by 3-ketosteroid 9α-hydroxylase (33), which, by a vinylogous retro-aldol reaction, cleaves the B-ring under aromatization of the A-ring. This step resembles the hydroxylation leading to **M3**, which was found to be a key step in the degradation of the substrates investigated here. However, in the current case, aromatization was not possible because of the *gem*-dimethyl moiety at C(4), which left ring cleavage under the formation of a linear C (5) side chain as the most straightforward option for productive degradation.

The genes required for the degradation pathway are not organized as an operon, unlike the other examples mentioned above, such as steroid metabolism (11), or the early degradation steps of camphor by the CamDCAB operon (34). Thus, this pathway might not have developed specifically as a functional unit for the metabolism of these substrates, and the ability to degrade these substrates might also be a secondary function of enzymes required for other metabolic traits. The fact that the genes are not organized in an operon has also hampered our efforts to identify the genes for the full metabolic sequence, which will require further research. However, the availability of the genome sequence and elucidation of the structure of key metabolites will facilitate such efforts.

The high stereospecificity of this degradation pathway is striking. Whereas analogs with a modified C-ring are readily degraded via this specific metabolic sequence, changing the configuration at one chiral center or presenting the substrates in their enantiomeric form prevents metabolization by the same bacterium. This specificity may contribute to the fact that it has proven challenging to develop dependable *in silico* models to predict biodegradation of complex, branched, or polycyclic substrates, as the presence of highly specialized microbial strains and their metabolic abilities in sewage treatment plants is still difficult to predict. Nevertheless, elucidating pathways for such complex substrates remains a pre-requisite to develop mechanistically based models for biodegradation, which are key in the rational design of biodegradable ingredients. This narrow substrate specificity of a biodegradation pathway for such complex natural substrates also supports initiatives to produce nature-identical compounds for industrial use, for example, using biotechnological approaches, as has been extensively studied in the case of (−)-Ambrox (30).

## Data Availability

All data are publicly available in the main text and supplemental material.

## SUPPLEMENTAL MATERIAL

Supplemental material aem.00876-26-s0001.pdf Detailed additional analytical data on the identified metabolites, structure elucidation by NMR, chemical synthesis, and genetic analysis.

ASM does not own the copyrights to Supplemental Material that may be linked to, or accessed through, an article. The authors have granted ASM a non-exclusive, world-wide license to publish the Supplemental Material files. Please contact the corresponding author directly for reuse.
