# Supplementary material for: A pathway for complete mineralization of the ambergris constituent (−)-Ambrox in Sphingomonas sp
Source: Appl Environ Microbiol. 2026 Aug 5;92(9):e00876-26. doi: 10.1128/aem.00876-26 (PMC13599519; doi:10.1128/aem.00876-26)
Supplement: Supplementary file 1 [file aem.00876-26-s0001.pdf]

Supporting Information to:

A pathway for complete mineralization of the Ambergris constituent (-)-Ambrox in *Sphingomonas sp.*

Tina Haupt, Felix Flachsmann, Gerhard Brunner, Yumiko Weiner-Sekiya, Lucas Hortencio, Cyrille Jarin, Patrick Robe, Alessio Fonzo, Francis Voirol, Eric Eichhorn, and Andreas Natsch

Contents

1. Isolation and structure elucidation of metabolites 2

2. General information for chemical synthesis of labelled substrates 18

2.1. Synthesis of [20-^{13}C]-(-)-Ambrox 18

2.2. Synthesis of [17-^{13}C]-(-)-Ambrox 20

3. Metabolite formation from labelled substrates, metabolite formation by different strains and from different substrates 22

4. Genetic analysis of early steps of the biosynthesis pathway 25

1. Isolation and structure elucidation of metabolites

a) LC MS analysis

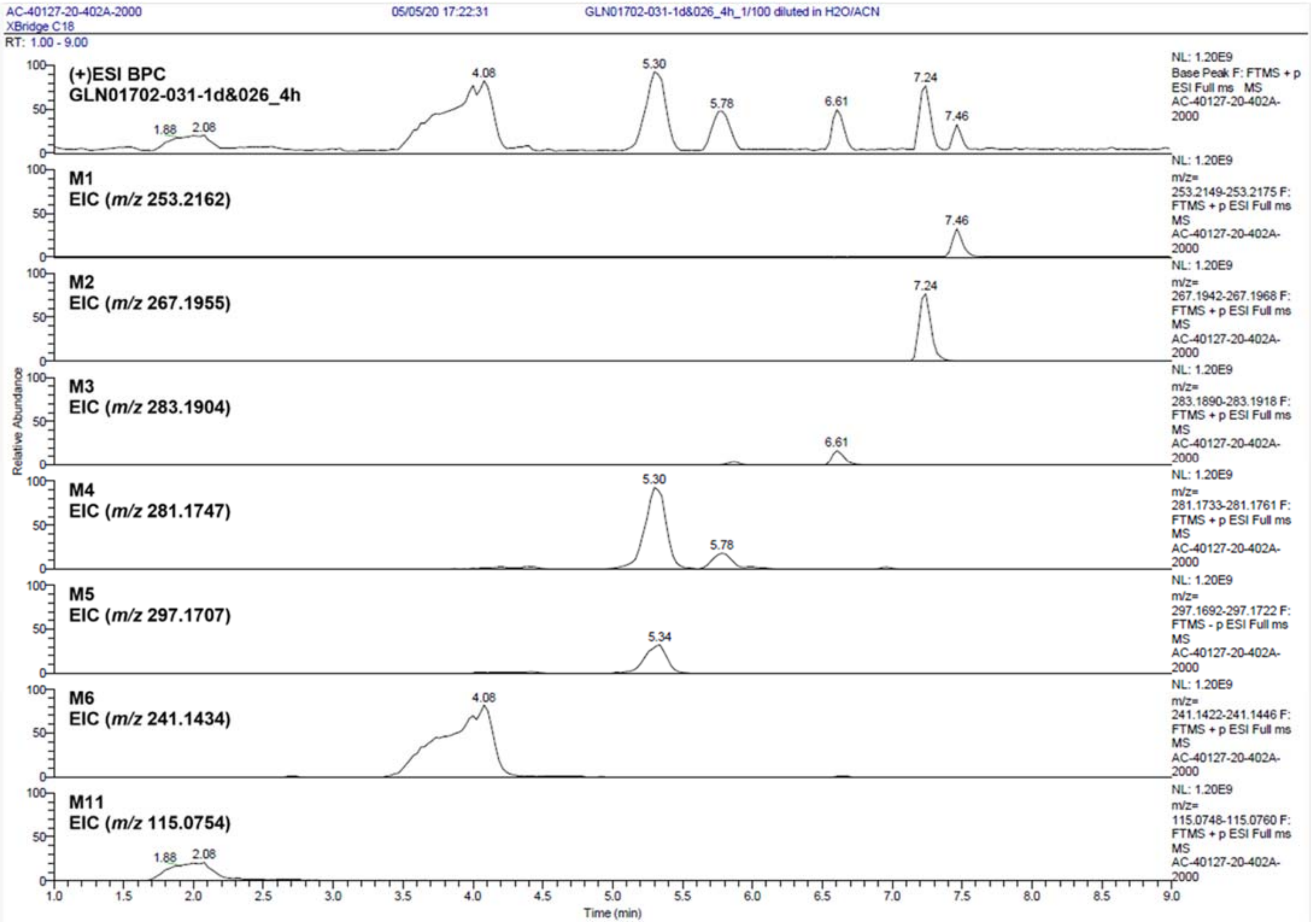

Figure S1. HPLC-HRMS analysis of the extract from incubation of (-)-Ambrox with a resting cell culture of Ambrox-5 used for metabolite isolation. Base peak chromatogram of the extract and single ion traces for the *m/z* ratio $[M+H]^+$ for M1-M4, M6 and M11 and $[M-H]^-$ for the metabolites M5, which were then isolated by preparative HPLC from this sample prior to NMR analysis.

b) **Figures on NMR structure elucidation - 2D-NMR Data for metabolites M1, M2, M3, M4, M5, M6 and M11:**

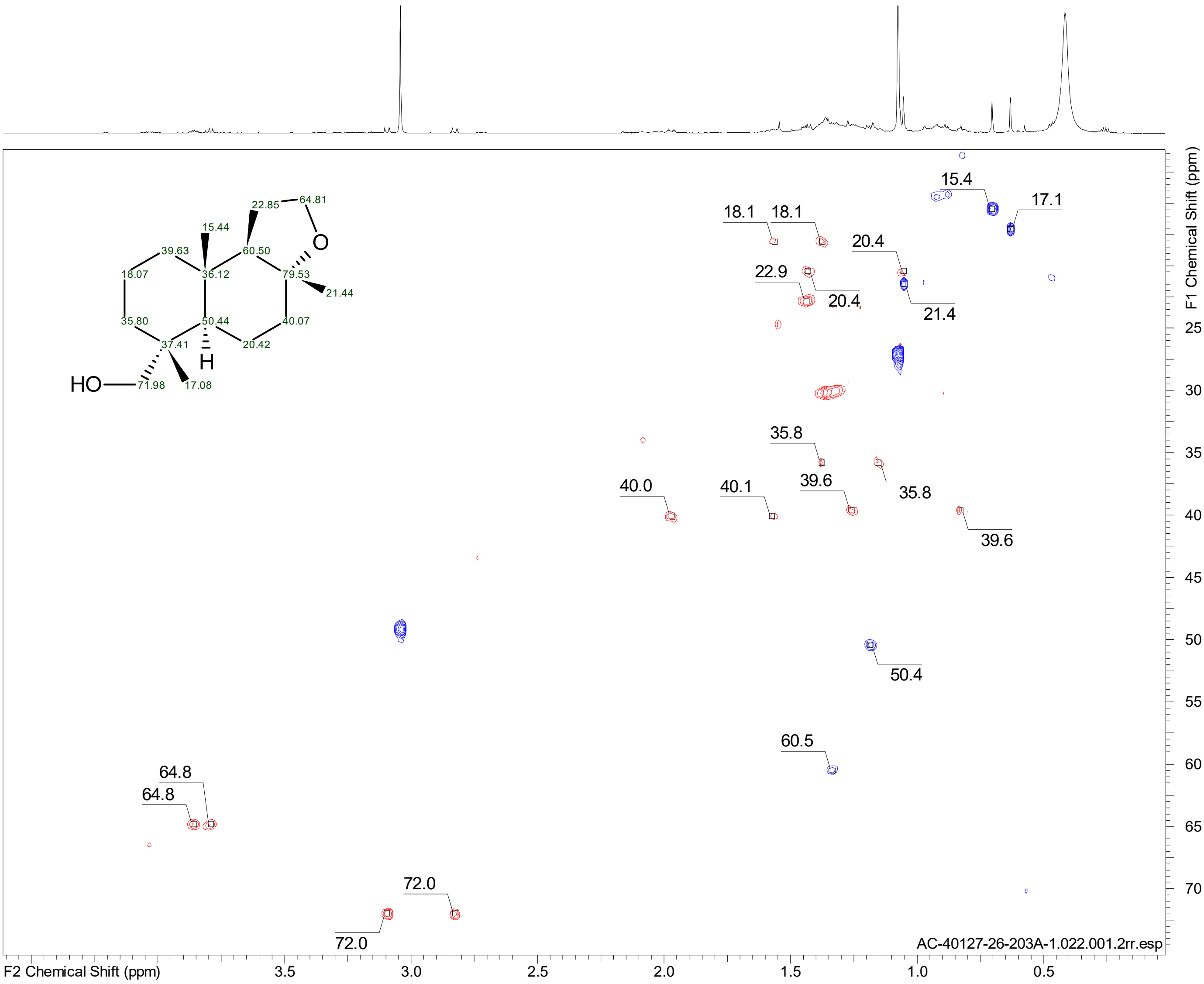

Figure S2. HSQC of M1. The two cross-peaks at 72.0 ppm show that a primary alcohol has been generated by oxidation of the former methyl group.

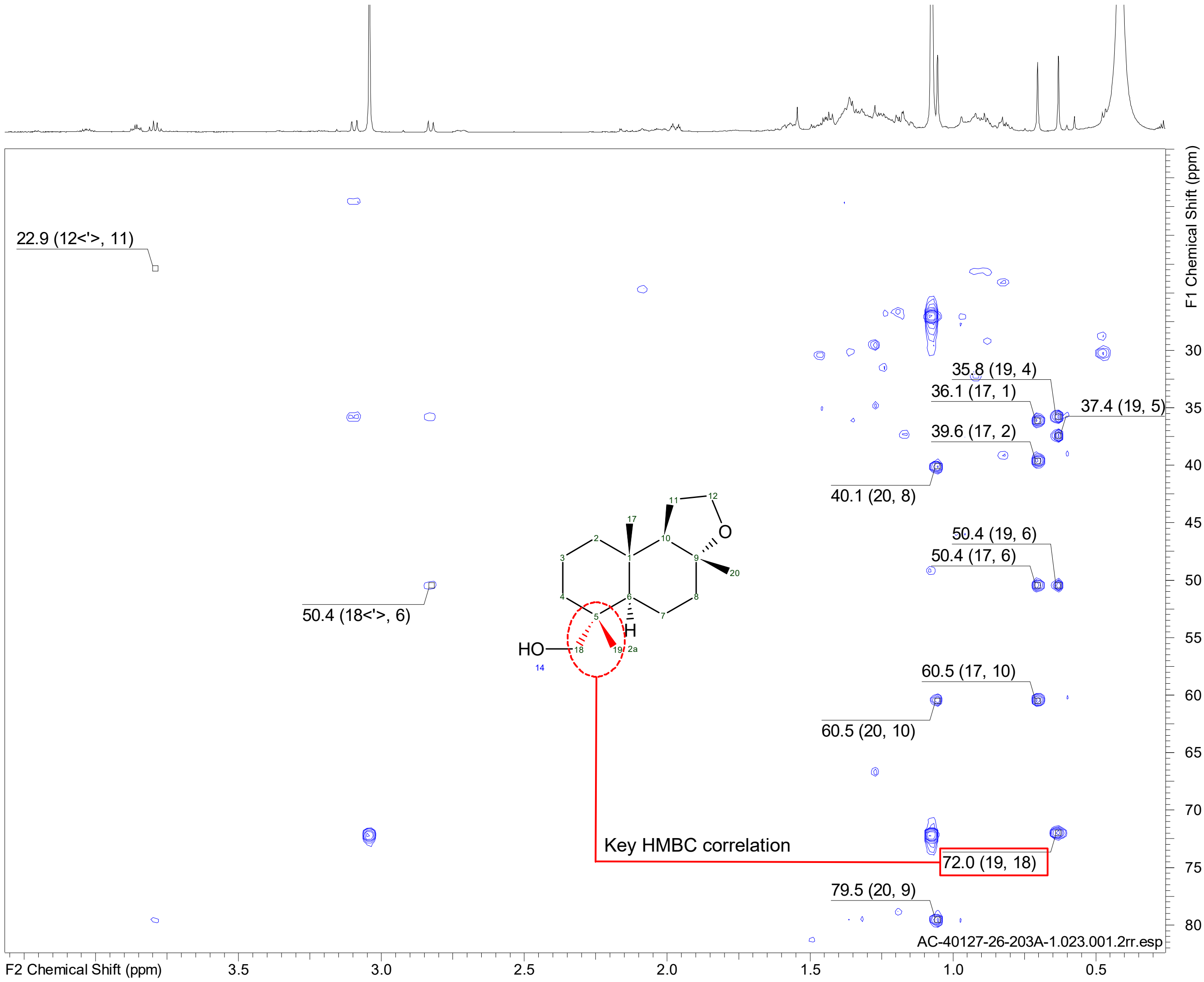

Figure S3. HMBC of M1. The primary alcohol C-18 at 72.0 ppm can be located by the key HMBC $^3J_{HC}$ correlation starting from the methyl H-19.

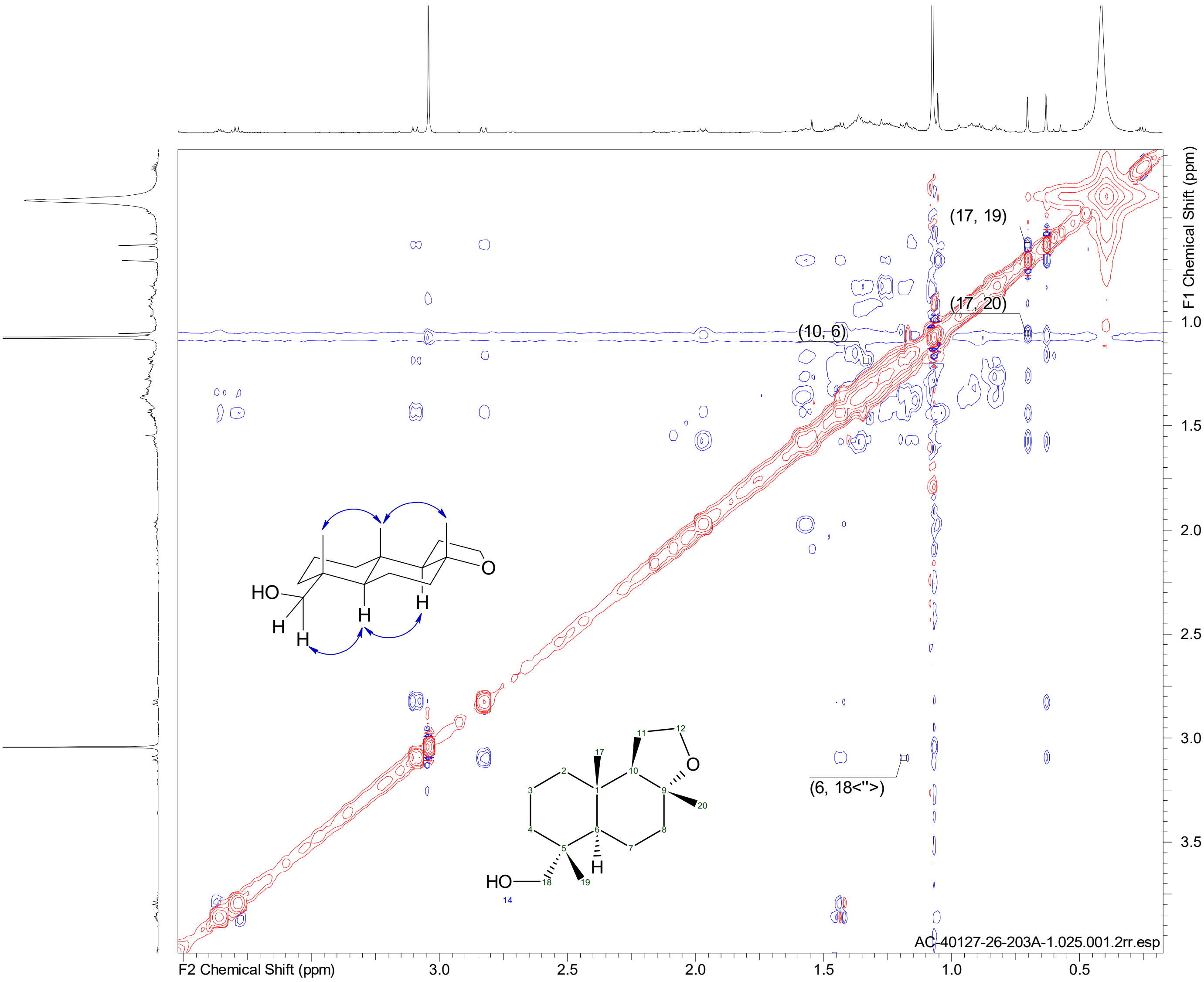

Figure S4. NOESY of M1. The selected NOESY cross-peaks confirm the relative configuration stemming from the parent compound Ambrox. In particular, the cross-peaks H-17/H-19 and H-6/H-18 indicate which of the *gem*-dimethyls has been oxidised, namely the equatorial one.

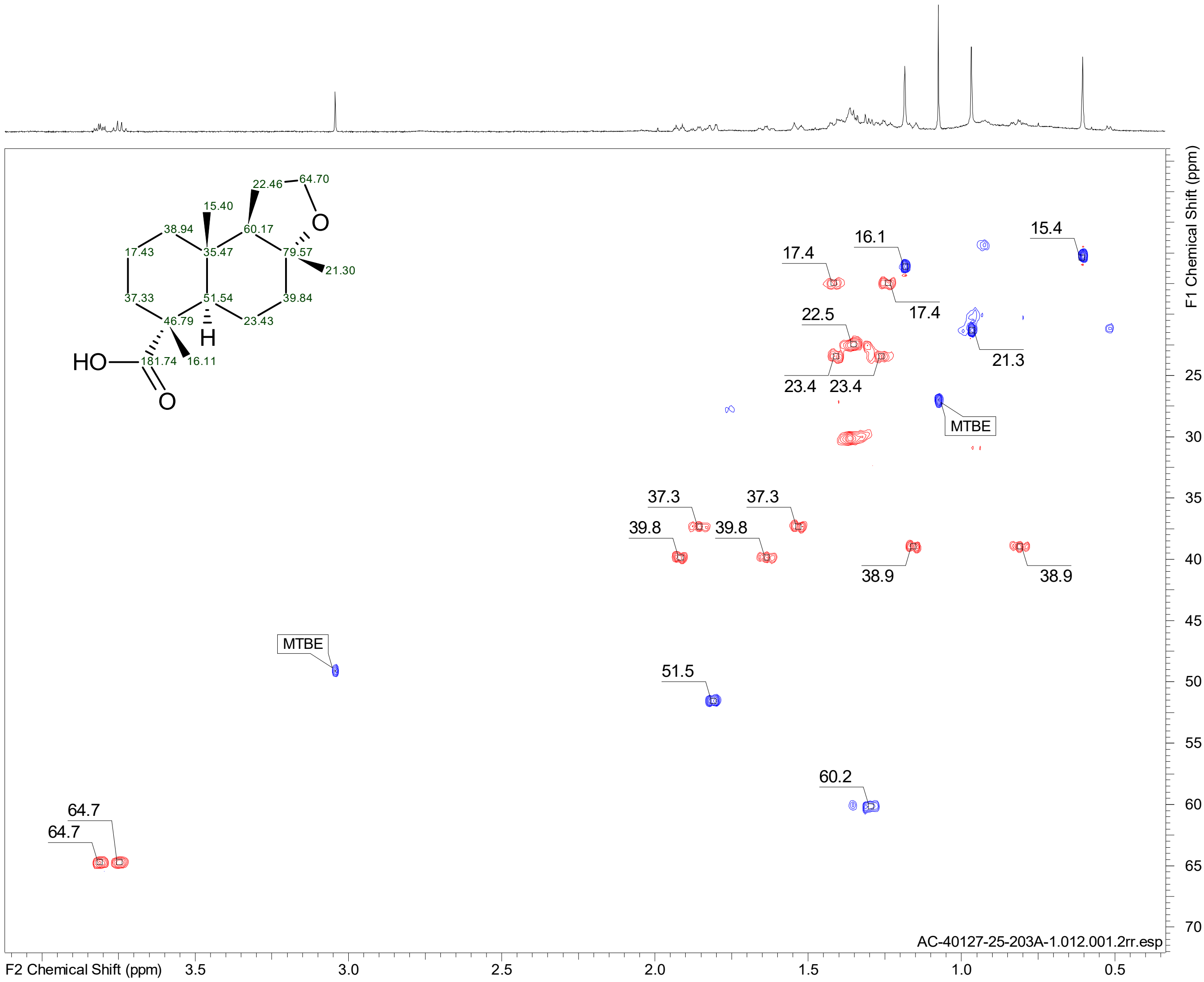

Figure S5. HSQC of M2.

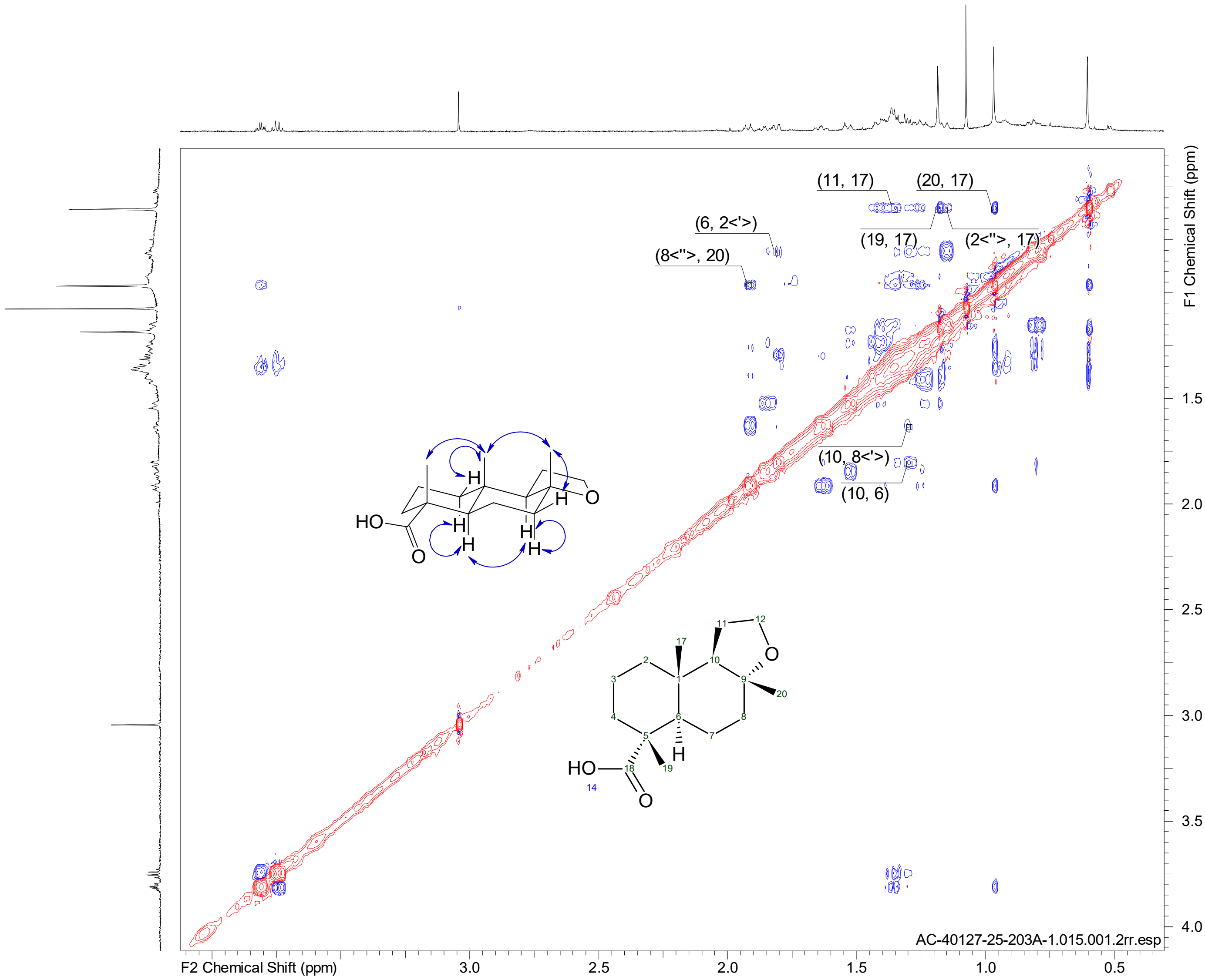

Figure S6. NOESY of M2. The selected NOESY cross-peaks confirm the relative configuration stemming from the parent compound Ambrox.

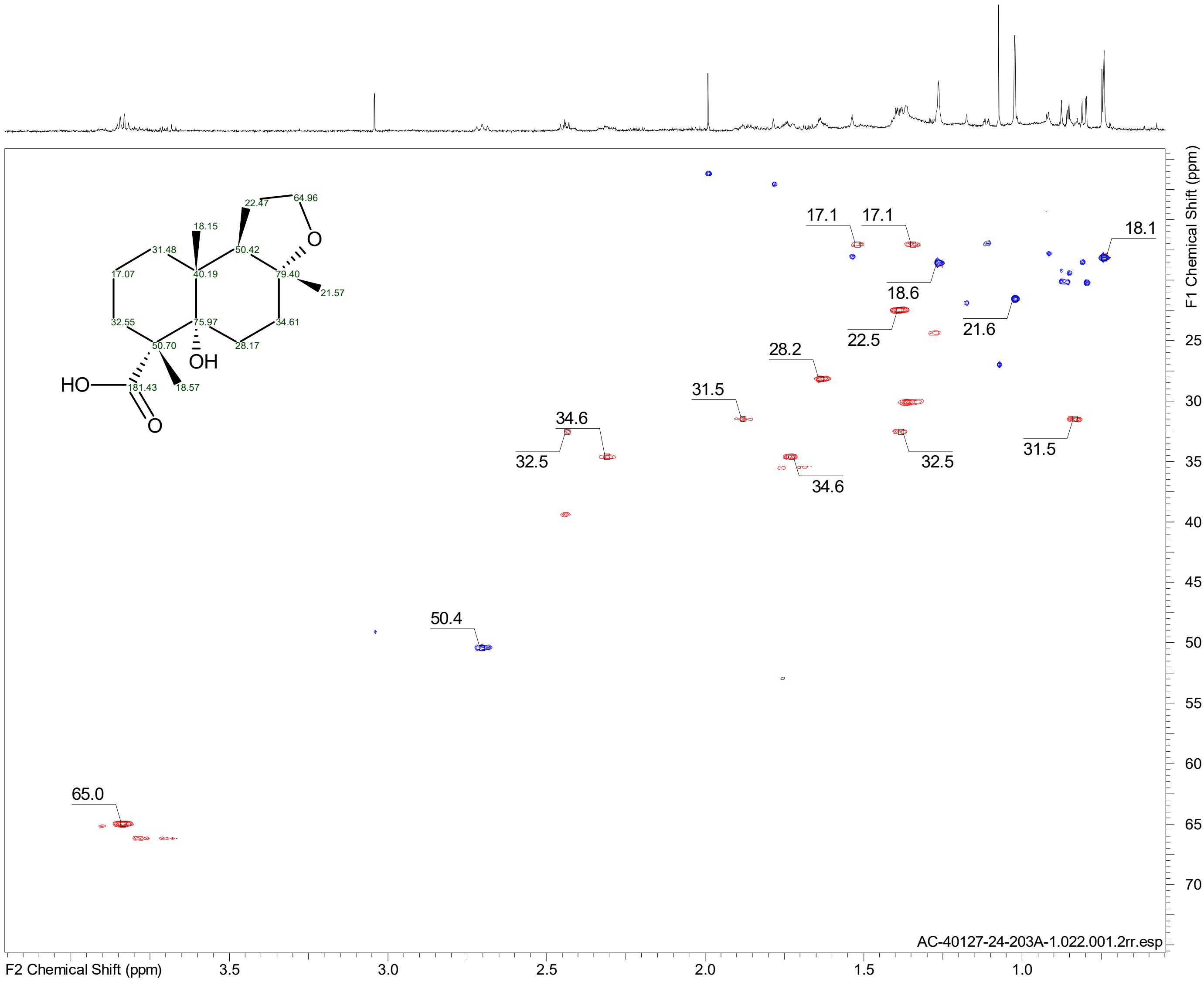

Figure S7. HSQC of M3. Compared to M1, M3 shows no more primary alcohol and neither the methine at the ring junction of cycles A and B.

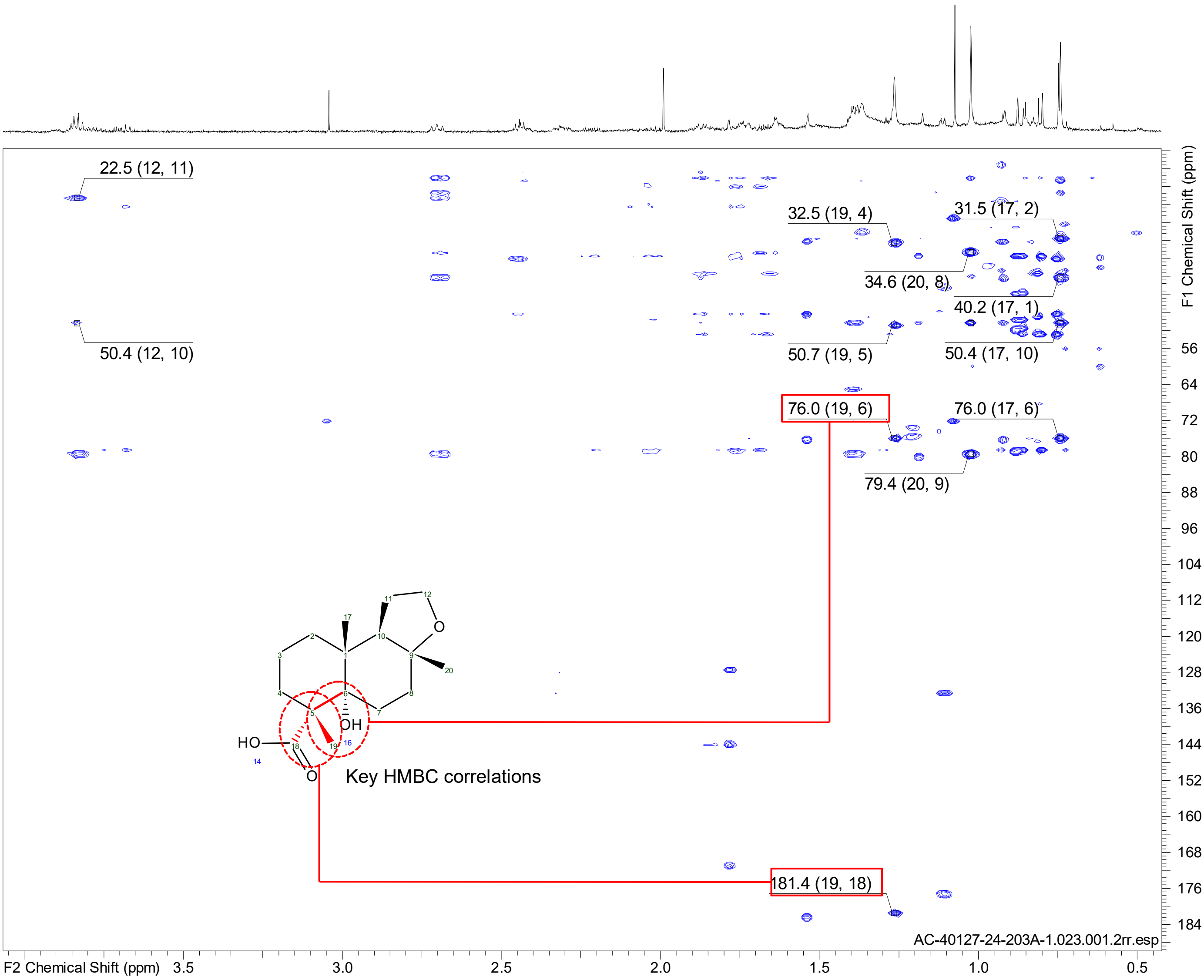

Figure S8. HMBC of M3. Starting from methyl H-19, two key HMBC $^3J_{HC}$ correlations locate the carboxylic acid C-18) as well as the tertiary alcohol (C-6) in the tricyclic scaffold.

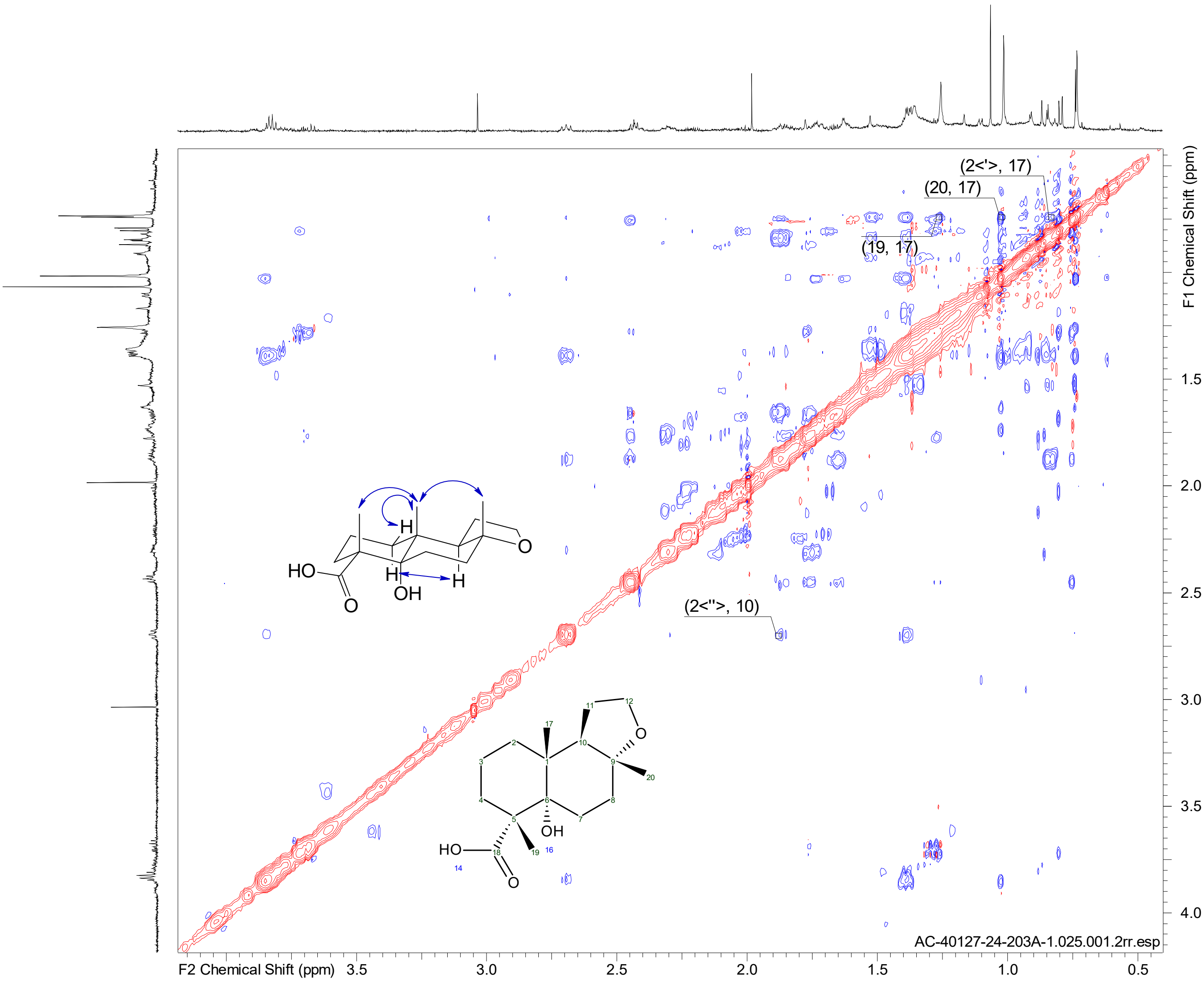

Figure S9. NOESY of M3. The selected NOESY cross-peaks confirm the relative configuration stemming from the parent compound Ambrox.

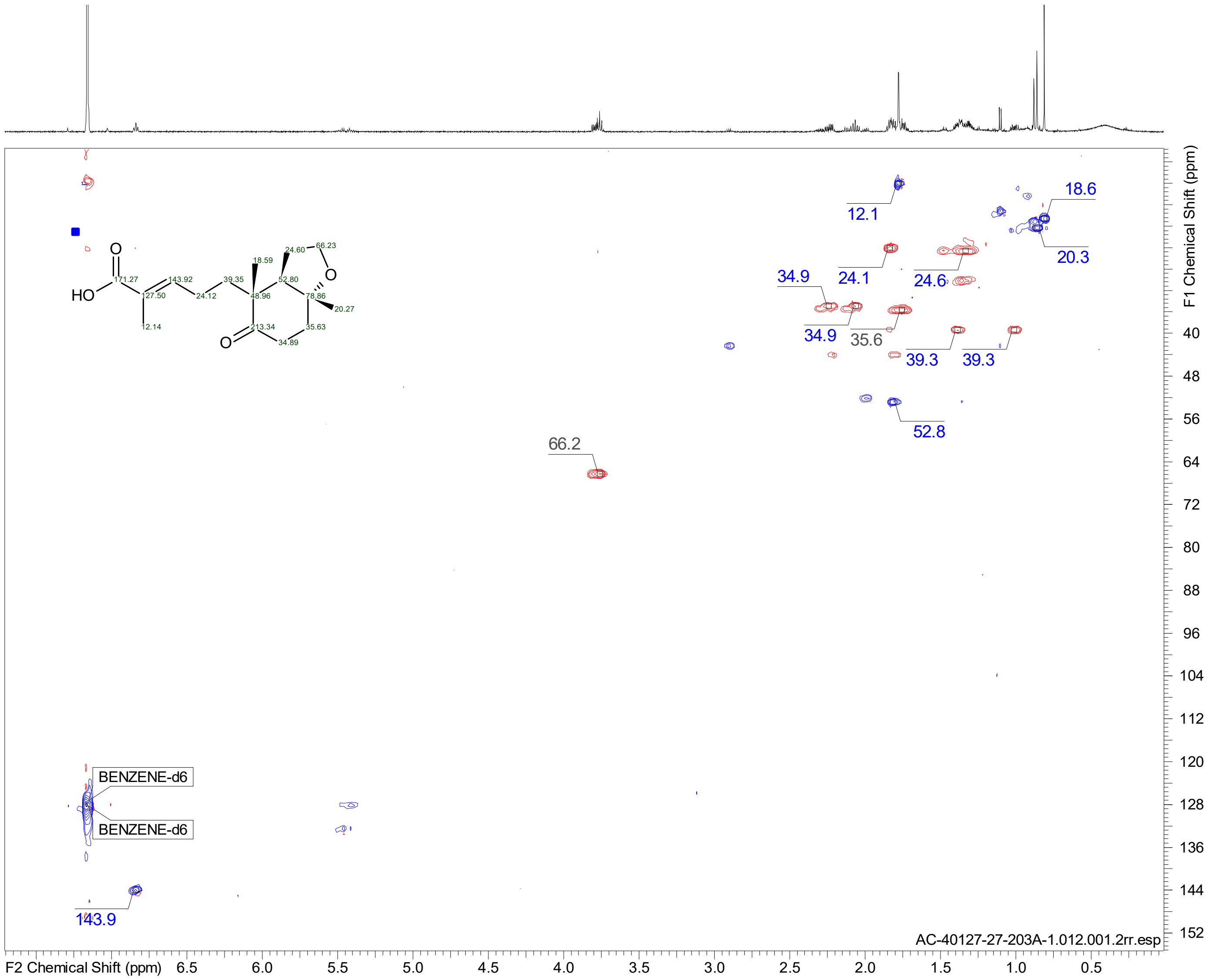

Figure S10. HSQC of M4. Compared to M3, M4 includes a double bond at 143.9 ppm, conjugated with the earlier discovered carboxylic acid. This suggests that the ring A has been opened.

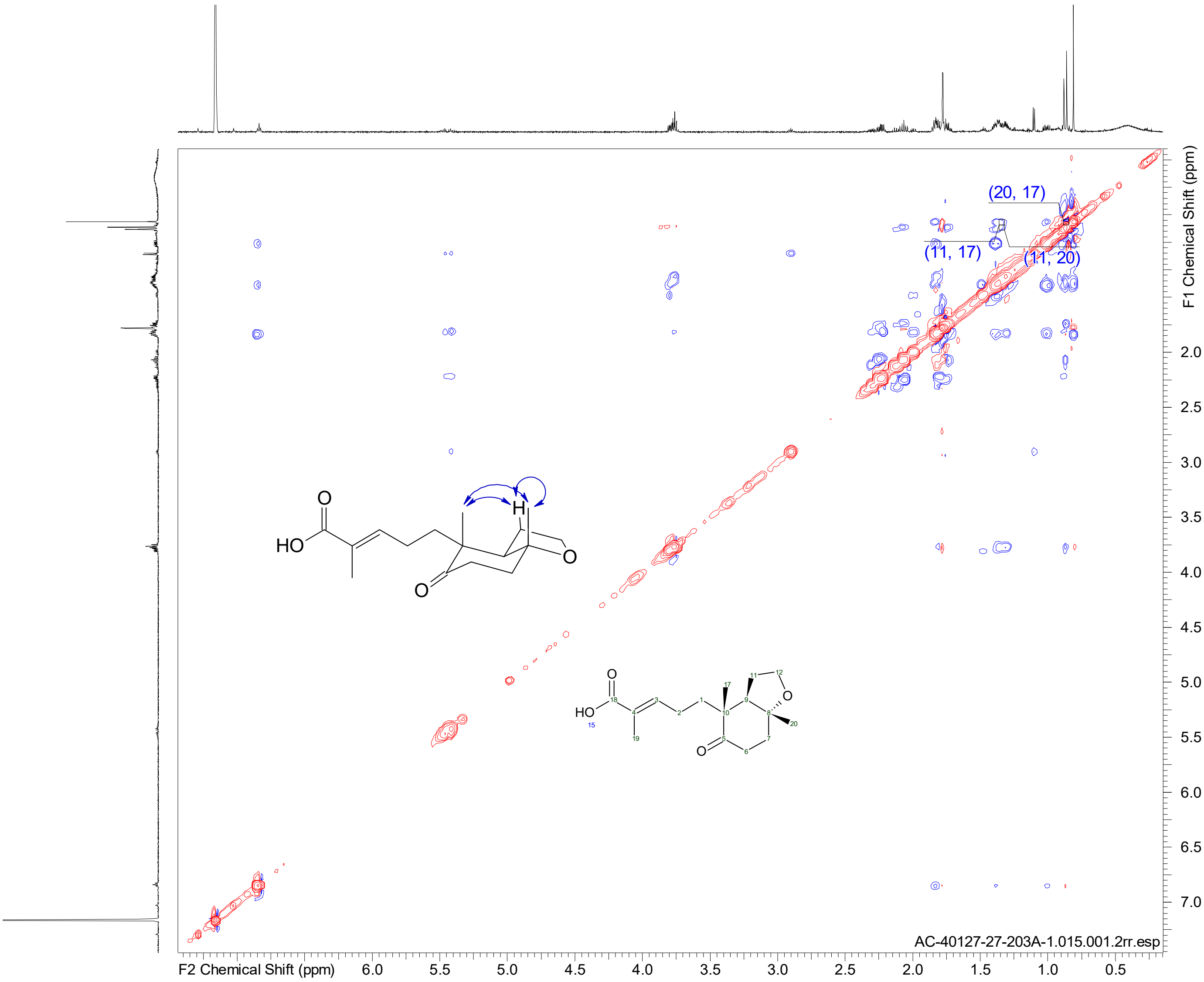

Figure S11. NOESY of M4. The selected NOESY cross-peaks confirm the relative configuration stemming from the parent compound (-)-Ambrox, at least for the remaining rings B and C.

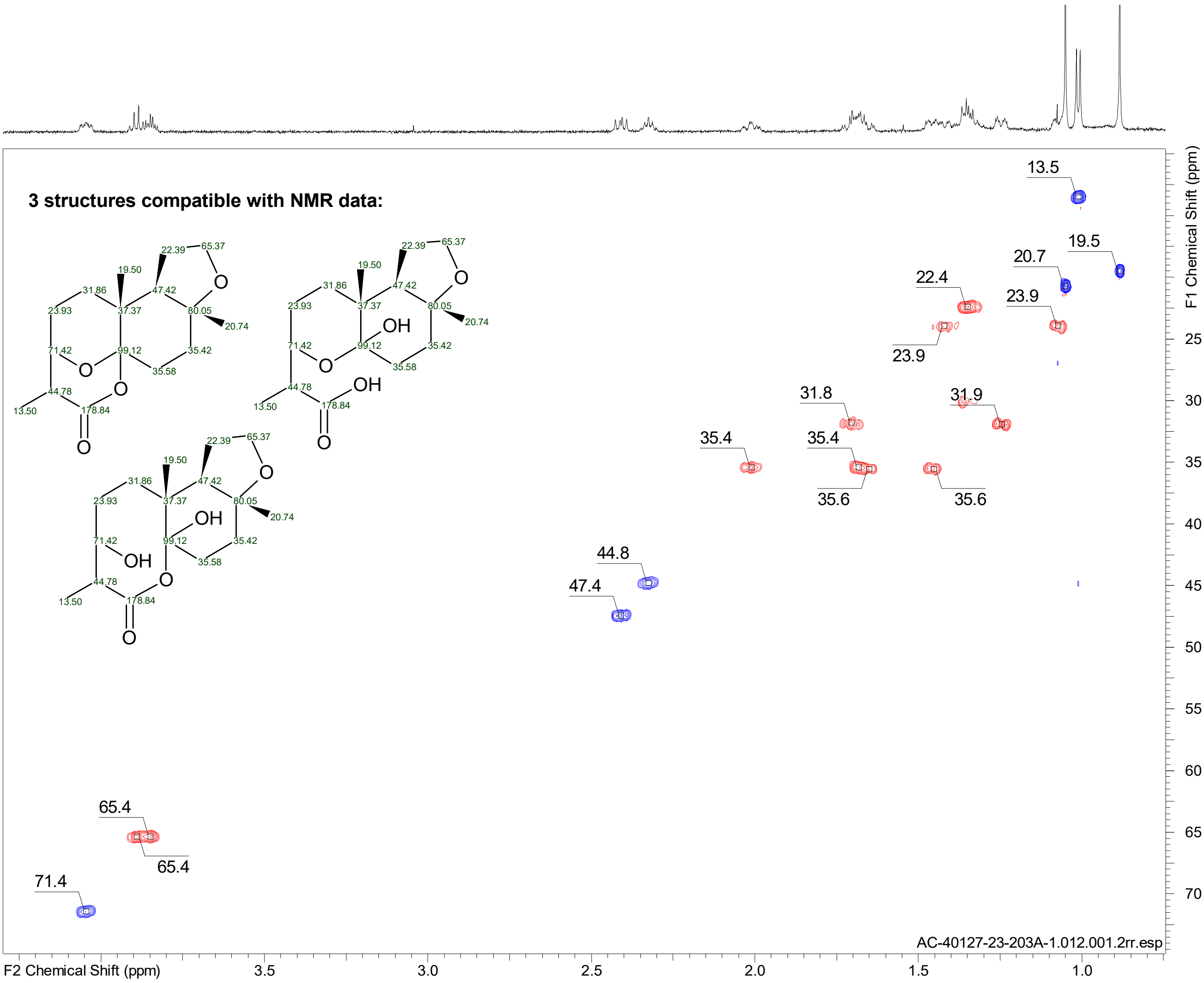

Figure S12. HSQC of M5. Depicted are three structural forms compatible with the available NMR data.

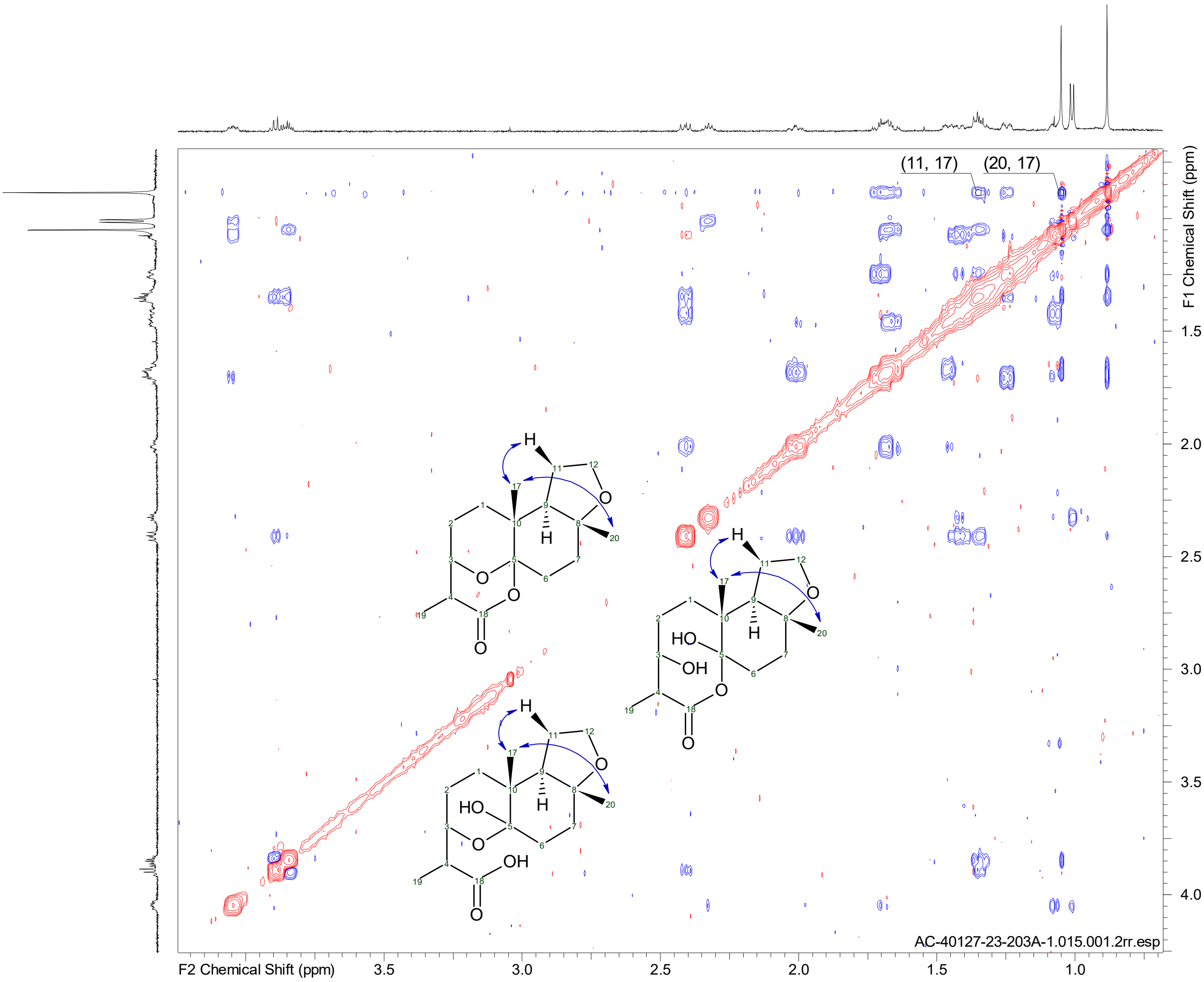

Figure S13. NOESY of M5. Depicted are three structural forms compatible with the available NMR data. The selected NOESY cross-peaks confirm the relative configuration stemming from the parent compound (-)-Ambrox, at least for the remaining rings B and C.

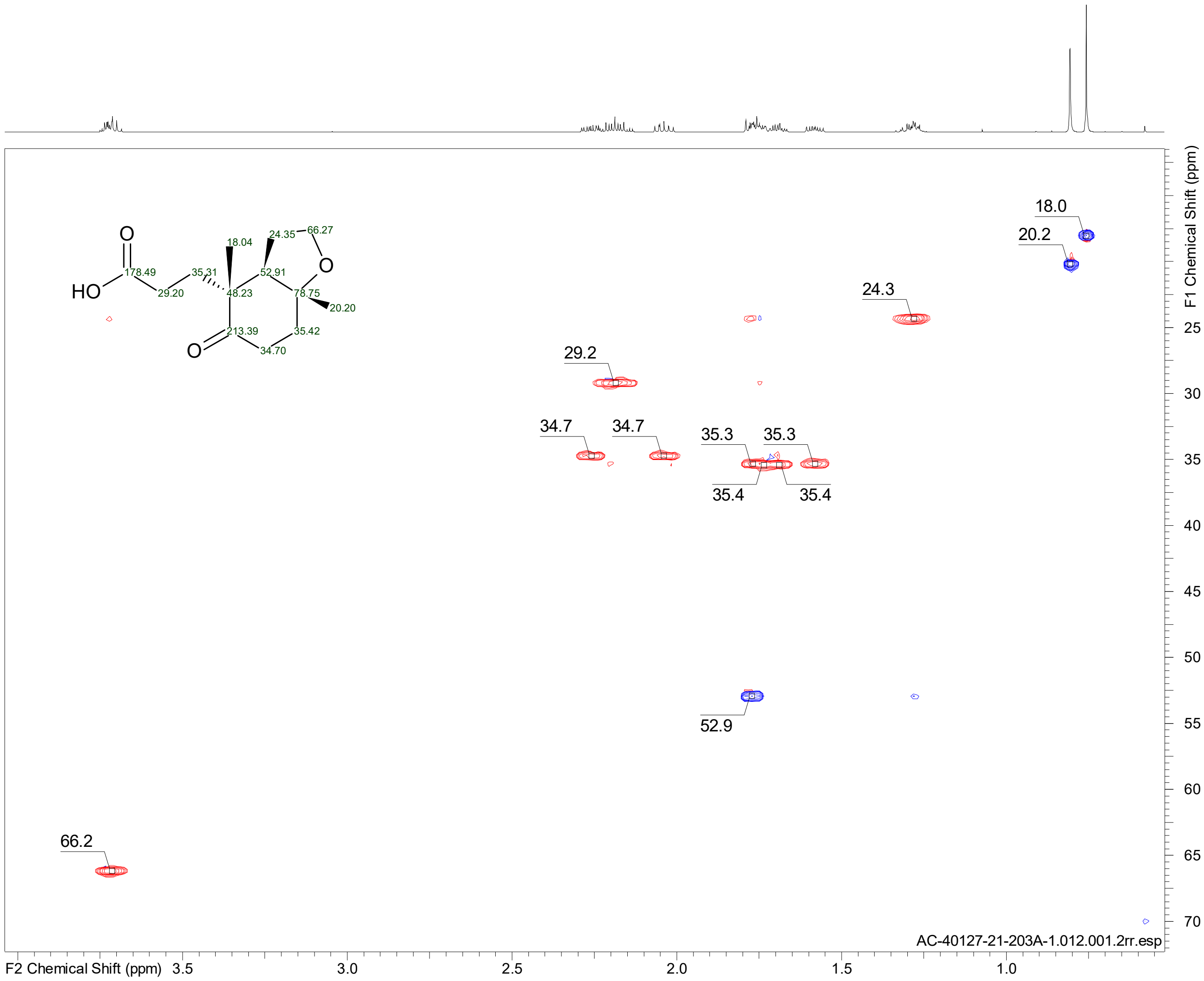

Figure S14. HSQC of M6.

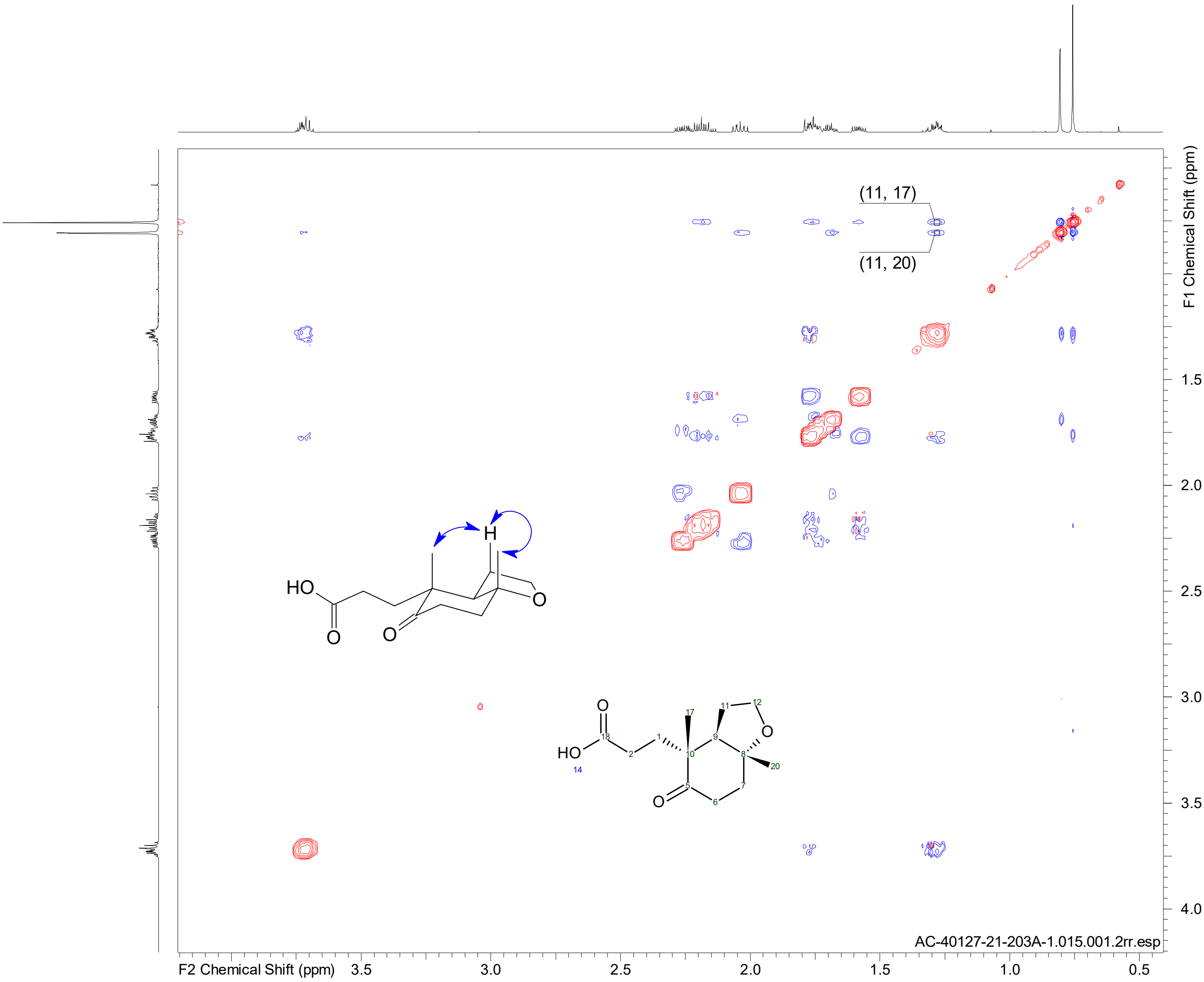

Figure S15. NOESY of M6. The selected NOESY cross-peaks confirm the relative configuration stemming from the parent compound Ambrox, at least for the remaining rings B and C.

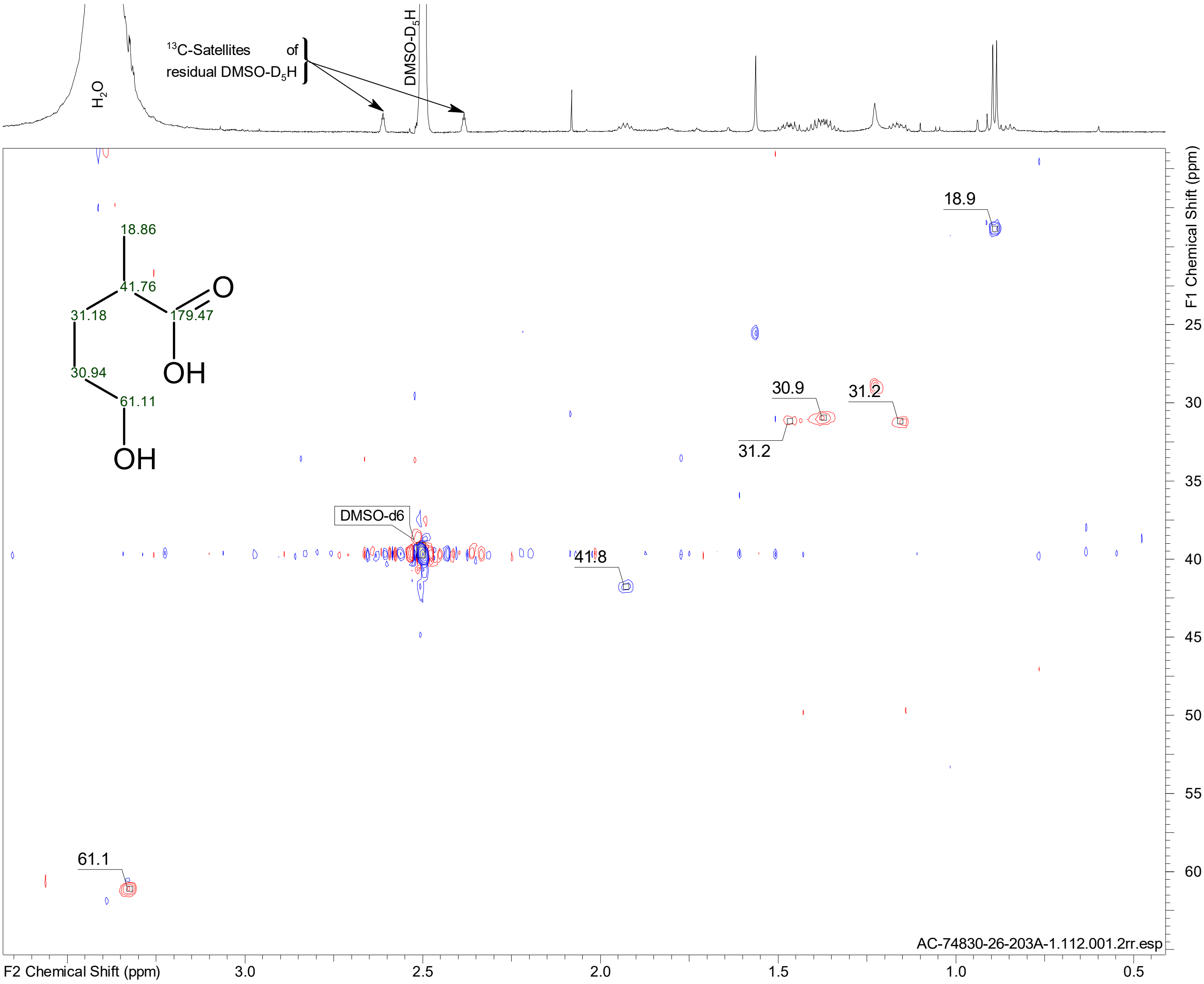

Figure S16. HSQC of M11 in DMSO-D6 (99.95% D).

2. General information for chemical synthesis of labelled substrates

All reagents and reaction solvents were analytical grade, purchased from commercial suppliers and used without further purification. Reactions were monitored by GC-FID (Zebron ZB-5 GC capillary column, 12 m x 0.32 mm x 0.25 µm, or Zebron ZB-wax, 15 m x 0.32 mm x 0.25 µm). Standard workup denotes washing organic layers to pH neutrality with brine, followed by drying the combined organic layers with $MgSO_4$, filtration and concentration in a rotary evaporator at 40-45°C under vacuum. Flash column chromatography (FC) was performed on Biotage silica gel prepacked columns (particle size 20 µm) with the indicated eluents, flow 50 mL/min. All reported yields, unless otherwise specified, refer to spectroscopically and chromatographically pure isolated compounds; isomeric ratios are indicated if appropriate. Routine NMR spectra were recorded on Bruker Avance III HD, 2D NMR spectra were recorded on Bruker Avance-III 600 MHz with 1.7 mm TCI-microcryoprobe. Proton chemical shifts are reported in ppm (δ) relative to tetramethylsilane (TMS), with the solvent resonance employed as the internal standard (CDCl3 δ 7.27 ppm). Data are reported as follows: chemical shift, multiplicity (s = singlet, d = doublet, t = triplet, q = quartet, p = pentet, sext = sextet, h = heptet, m = multiplet, br = broad), coupling constants (Hz) and integration. ^{13}C chemical shifts are reported in ppm from tetramethylsilane (TMS) with the solvent resonance as the internal standard (CDCl3 δ 77.0 ppm). Mass spectra were recorded with GC-MS (Agilent Technologies 7890A / 5975C) equipped with an SGE BPX5 capillary column (12 m, 0.22 mm i.d., 0.25 µm film) operated at a constant He flow of 1 mL/min. The temperature program started at 50 °C (for 2 min), then with 20 °C/min to 240 °C followed by 35 °C/min to 270 °C (for 3 min). The MS transfer line and ion source temperatures were 250 °C and 230 °C, respectively. The quadrupole MS was equipped with an EI ion source at 70 eV. For high-resolution mass spectra, samples were diluted in methanol and directly introduced in the MS (Thermo Scientific, Q Exactive Orbitrap) by a syringe pump (Chemyx Inc., Fusion 100 T) at a flowrate of 5-10 µL/min. Data was recorded with 70.000 mass resolution using Xcalibur (4.1.50) and analyzed with Xcalibur (4.2.28.14).

2.1. Synthesis of [20-^{13}C]-(-)-Ambrox

2.1.1. 8,12-dimethyl-4-(methyl-^{13}C)trideca-3,7,11-trien-1-ol

a) The freshly prepared solution of $^{13}CH_3$-MgI (0.7 M in Et_2O, 84 mL, 58.8 mmol, 1.1 equiv.) was added dropwise to the cooled (10°C) solution of 5,9-dimethyldec-4-enal (E/Z-mixture, 10.0 g, 55.5 mmol, 1 equiv.) in Et_2O (100 mL). After finished addition (temperature 26°C) stirring was continued for 10 min at RT. The mixture was poured on ice cold 2M aqueous HCl solution (80 mL) and extracted with MTBE (150 mL). After usual workup, (1-^{13}C)-6,10-dimethylundeca-5,9-dien-2-ol was obtained as clear, yellow oil (8.4 g, 77%, E/Z = 57:43).

b) The crude product (8.4 g, 43 mmol, 1 equiv.) was dissolved in CH_2Cl_2 (150 mL) and pyridinium chlorochromate (13.0 g, 60 mmol, 1.4 equiv.) was added in one portion. The mixture was stirred for 2 h upon which the temperature rose to 30°C. After filtration by suction over silica gel and rinsing the filter cake with MTBE (150 mL), the solvent was evaporated o yield (1-^{13}C)- 6,10-dimethylundeca-5,9-dien-2-one-as a clear, green-brown liquid (90%, E/Z = 58:42).

c) The crude product (7.5 g, 38 mmol, 1 equiv.) was dissolved in THF (150 mL) and the solution was cooled to 10°C. The solution of vinyl magnesium bromide (0.7 M in THF, 74 mL, 51.8 mmol, 1.4 equiv.) was added dropwise during 15 min, upon which the temperature rose to 28°C. Stirring at RT was continued for 20 min, then the mixture was poured on ice cold saturated aq. NH4Cl solution (200 mL) and acidified by addition of 2 M aq. HCl solution (20 mL). After usual workup, 3-(methyl-^{13}C)-7,11-dimethyldodeca-1,6,10-trien-3-olwas obtained as clear, yellow liquid (7.70 g, 90%, E/Z = 58:42).

d) The crude product (7.50 g, 34 mmol) and 1,10-phenanthroline (10 mg) were dissolved in THF (100 mL). The solution was cooled to 0°C and butyl lithium (1.6 M solution in hexane, 21.2 mL, 34 mmol, 1.0 equiv.) was added dropwise (colour change from yellow to dark brown). After completed addition, the cooling bath was removed and stirring continued for 10 min, then methyl chloroformate (3.8 g, 40 mmol, 1.2 equiv.) was added dropwise. The mixture was stirred for 30 min at RT, then poured on cold sat. aq. NH_4Cl solution, then acidified by addition of 2M aq. HCl-solution (10 mL). Standard workup yielded 3-(methyl-^{13}C)-7,11-dimethyldodeca-1,6,10-trien-3-yl methyl carbonate as a clear colourless liquid (8.8 g, 94%).
e) The crude product (8.8 g, 31 mmol, 1 equiv.) was dissolved in EtOH (70 mL) and $Pd(OAc)_2$ (280 mg, 1.3 mmol, 4 mol%), sodium propionate (1.2 g, 13 mmol, 0.4 equiv.) and triphenyl phosphine (0.98 g, 3.8 mmol, 0.12 equiv.) were added. The mixture was placed in a stainless-steel autoclave and stirred at RT under 40 bar carbon monoxide for 22 h. After standard workup methyl 4-(methyl-^{13}C)-8,12-dimethyltrideca-3,7,11-trienoate (9.0 g, >90%) was obtained as a brown, viscous liquid.
f) The crude product (9.0 g, 34 mmol, 1 equiv.) was dissolved in Et_2O (50 mL) and the solution was added dropwise during 20 min to the suspension of $LiAlH_4$ (1.2 g, 31 mmol, 0.9 equiv.), during which the solution started to reflux. The mixture was stirred at RT for 30 min, then poured carefully on ice cold 2M aq. HCl-solution (100 mL). Standard workup furnished a clear, brown liquid which was purified by flash column chromatography (heptane/MtBE 84:16) to yield 4-(methyl-^{13}C)-8,12-dimethyl-trideca-3,7,11-trien-1-ol as a clear, slightly yellow liquid (1.7 g, 21% (6 steps), 4 isomers, 3*Z*,7*Z*:3*E*,7*Z*:3*Z*,7*E*:3*E*,7*E* = 14:21:24:36, purity 95% (sum of isomers)).
^{1}H-NMR (CDCl3, 400 MHz, mixture of E/Z isomers) δ ppm 5.03 - 5.21 (m, 3 H), 3.62 (t, *J*=6.5 Hz, 2 H), 2.25 - 2.34 (m, 2 H), 1.95 - 2.16 (m, 7 H), 1.88 - 1.92 (m, 1 H), 1.82 (br. s, 1 H), 1.70 (br. s, 6H), 1.63 (s, 3H), 1.62 (s, 3H).
^{13}C-NMR (CDCl3, 101 MHz) δ ppm 23.54 (3*Z*), 16.19 (3*E*).
GC-MS (EI, 70eV): 3*E*,7*E*-isomer (9.34 min): 237 (<1, M$^+$), 222 (<1), 207 (4), 194 (2), 168 (3), 153 (3), 136 (17), 124 (12), 108 (7), 95 (10), 81 (31), 69 (100), 53 (9), 41 (42), 31 (7).

2.1.2. [20-^{13}C]-(-)-Ambrox

The AacSHC* biocatalyst (Flachsmann, Aeberli et al. 2024) used for the cyclization of *E*,*E*-8,12-dimethyl-4-(methyl-^{13}C)trideca-3,7,11-trien-1-ol (36% in mixture prepared above under 2.1f) to [20-^{13}C]-(-)-Ambrox was produced as described elsewhere (Eichhorn, Locher et al. 2018) from *E. coli* BL21(DE3) cells transformed with a pET-28a(+)-based plasmid where the gene coding for *Aacshc** is under the control of an IPTG-inducible promoter. Briefly, 428 mg of substrate were incubated with *E. coli* AacSHC* cells (30 g/L *E*,*E*-isomer, 60 g/L cells) in a total volume of 4 mL at 45°C and pH 5.6 in presence of 0.054% sodium dodecyl sulphate for 51 h, resulting in 90% conversion of the *E*,*E*-isomer. The reaction was extracted 4x with 5 mL MtBE, whereafter the crude product was purified by FC (heptane/MtBE 19:1 to 9:1) to isolate [20-^{13}C]-(-)-Ambrox (14 mg, >99% pure) as a white solid besides recovered starting material (139 mg, 32%) and mixed product fractions.
GC-MS (EI, 70eV): 237 (2, M$^+$), 221 (100), 137 (36), 125 (11), 109 (11), 97 (45), 81 (21), 69 (20), 55 (27), 41 (34). HSQC-DEPT (C6D6) see Figure S17.

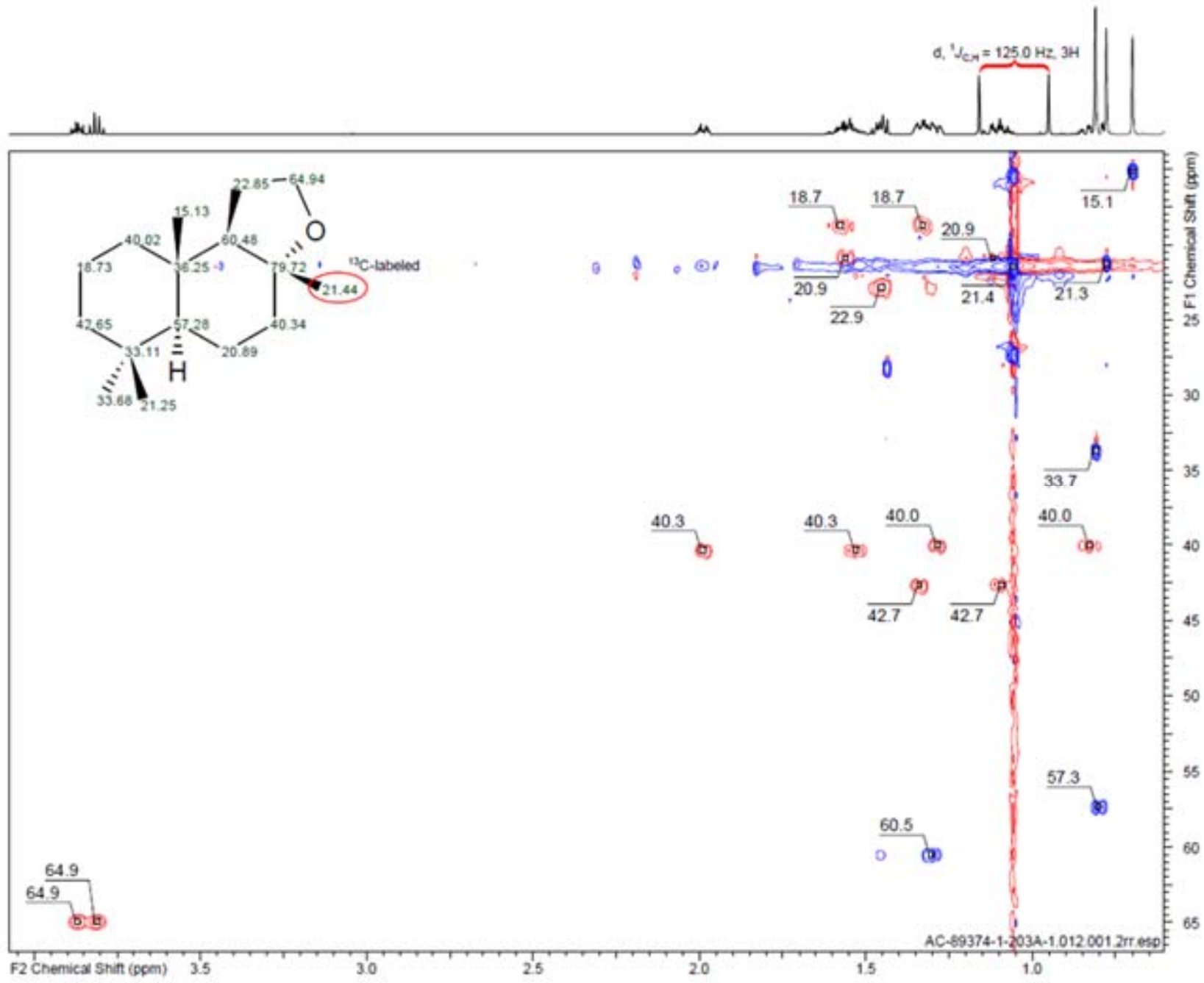

Figure S17. HSQC-DEPT (C6D6) of [20-^{13}C]-(-)-Ambrox

2.2. Synthesis of [17-^{13}C]-(-)-Ambrox

2.2.1. 8-(methyl-^{13}C)-4,12-dimethyltrideca-3,7,11-trien-1-ol

a) Steps 1a-c) were repeated with 5-methylhex-4-enal (8.0 g, 71 mmol) to isolate 3-(methyl-^{13}C)-7-methylocta-1,6-dien-3-ol as a clear, yellow liquid (8.9 g).

b) The solution of crude product (4.0 g, 26 mmol, 1 equiv.) in 2-ethoxyprop-1-ene (8.9 g, 100 mmol, 4 equiv.) was placed in an autoclave and, triethyl amine (1 drop) and conc. phosphoric acid (1 drop) were added. The mixture was stirred at 150°C for 3 h in the closed autoclave, then concentrated in a rotary evaporator and the residue, a clear, dark brown liquid (3.4 g) was purified by FC (heptane/MtBE 30:1) to furnish 6-(methyl-^{13}C)-10-methylundeca-5,9-dien-2-one as a clear, yellow liquid (1.65 g, 33%, purity 87%, E/Z 51:36).

c) Steps 1c-f) were repeated with the above product to yield, after FC, the title compound as a clear, slightly yellow liquid (0.5 g, 30% (4 steps), 4 isomers, 3*Z*,7*Z*:3*E*,7*Z*:3*Z*,7*E*:3*E*,7*E* = 15:21:24:34, purity 94% (sum of isomers)).

^{1}H-NMR (CDCl3, 400 MHz, mixture of E/Z isomers) δ ppm 5.07 - 5.20 (m, 3 H), 3.63 (td, *J*=6.5, 1.7 Hz, 2 H), 2.27 - 2.34 (m, 2 H), 1.96 - 2.16 (m, 9 H), 1.86 (d, *J*=1.2 Hz, 1 H), 1.76 - 1.81 (m, 1 H), 1.73 - 1.76 (m, 1 H), 1.70 (d, *J*=1.2 Hz, 3 H), 1.64 - 1.68 (m, 2 H), 1.62 (d, *J*=3.9 Hz, 3 H), 1.55 (d, *J*=1.2 Hz, 1 H).

^{13}C-NMR (CDCl3, 101 MHz) δ ppm 23.36, 16.01, 16.00.

GC-MS (EI, 70eV): 3*E*,7*E*-isomer (9.34 min): 237 (<1, M^{+}), 194 (1), 168 (2), 153 (2), 137 (14), 124 (13), 108 (5), 94 (8), 82 (27), 69 (100), 53 (7), 41 (42).

2.2.2. [17-^{13}C]-(-)-Ambrox

A first SHC-mediated polycyclization attempt as described in chapter 2.1.2) with 368 mg 8-(methyl-^{13}C)-4,12-dimethyl-trideca-3,7,11-trien-1-ol prepared under 3.1) resulted in only 15% conversion of the *E,E*-isomer after 51 h. The substrate was recovered and repurified by FC. Successful SHC-mediated polycyclization was then carried out with 48 mg substrate and 156 mg *E. coli* AacSHC* cells (6 g/L substrate, 39 g/L cells) in a total volume of 4 mL at 45°C and pH 5.6 in presence of 0.21 % SDS, resulting in >99% conversion of the *E,E*-isomer in 24 h. The reaction was extracted 3x with 10 mL MtBE, whereafter the crude product was purified by FC (heptane/MtBE 19:1 to 9:1) to isolate [17-^{13}C]-(-)-Ambrox (9 mg, 97% pure) as a white solid.

GC-MS (EI, 70eV): 237 (2, M^+), 222 (100), 138 (48), 125 (10), 110 (11), 97 (54), 82 (16), 69 (21), 55 (25), 43 (42).

HSQC-DEPT (C_6D_6) see Figure S18.

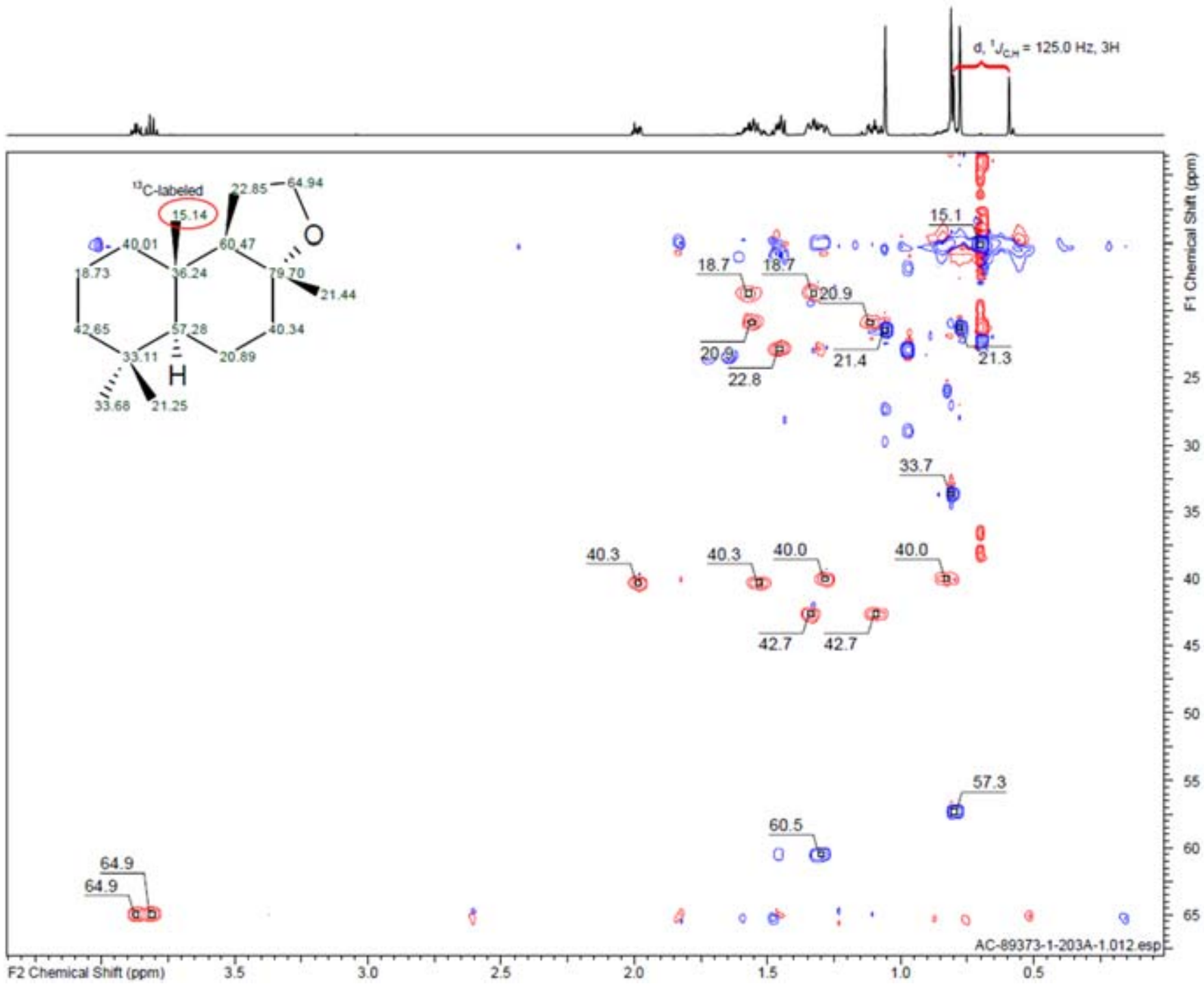

Figure S18. HSQC-DEPT (C6D6) of [17-^{13}C]-(-)-Ambrox

3. Metabolite formation from labelled substrates, metabolite formation by different strains and from different substrates

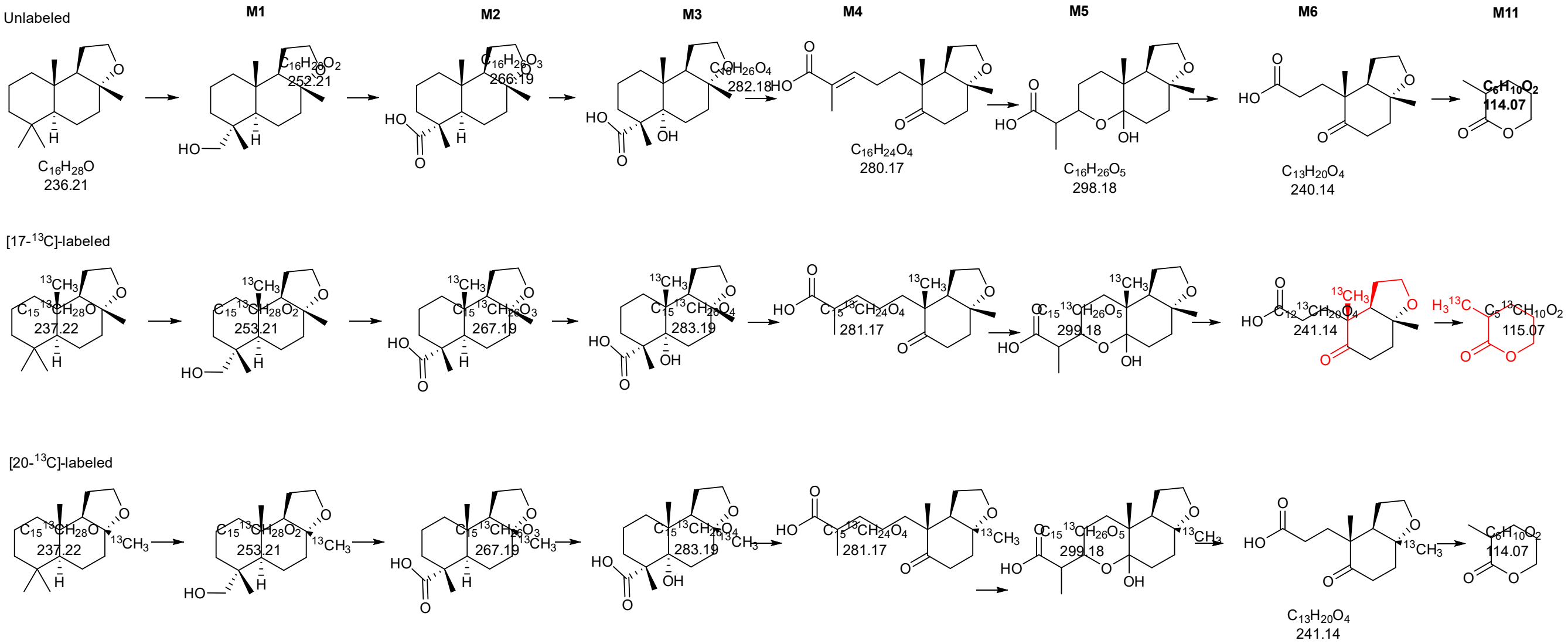

Figure S19. More details related to Figure 4 in main manuscript. Metabolites observed by HPLC-HRMS after incubation of unlabelled, and [17-^{13}C]- and [20-^{13}C]-labeled (-)-Ambrox with strain Ambrox-5 for 4 h – 2 days. Shown are all the exact massed observed and the metabolites associated with the exact mass, indicating that only M11 originating from [17-^{13}C] labelled substrate retains the initial ^{13}C -label, while M1 – M6 retain the label for both labelled substrates. The positioning of **M10*** subunit in **M6** of[17-^{13}C]-(−)-Ambrox is shown in red. The corresponding masses were detected at all three time points.

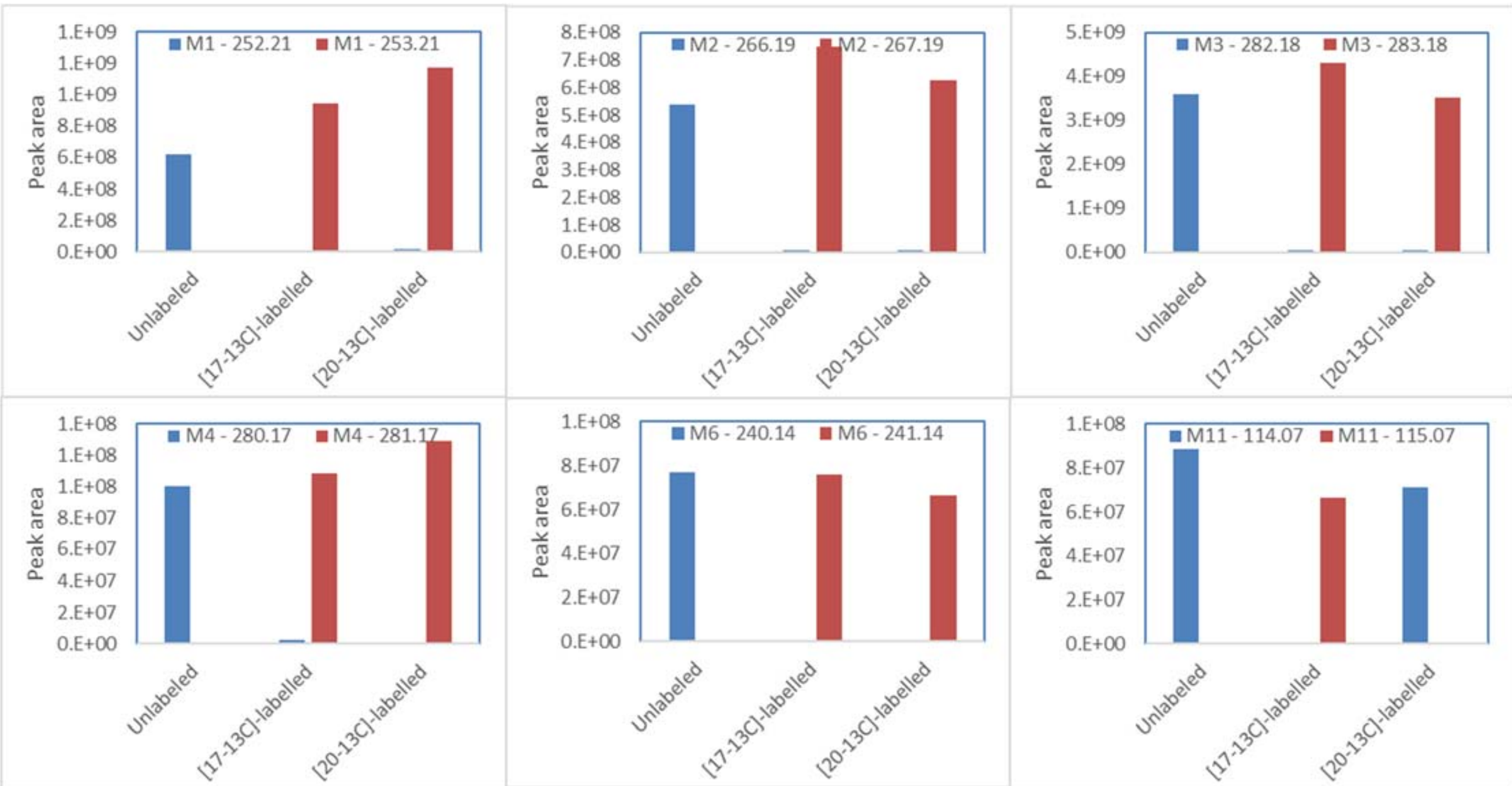

Figure S20. Quantitative data related to Figure 4 in main manuscript. Metabolites observed by HPLC-HRMS after incubation of unlabelled, and [17-^{13}C]- and [20-^{13}C]-labeled (-)-Ambrox with strain Ambrox-5 for 4 h – 2 days. Shown are peak areas of the unlabelled metabolites (blue) and labelled metabolites (red) with their respective isotopic masses after 4 h. These metabolites were observed at all three time points.

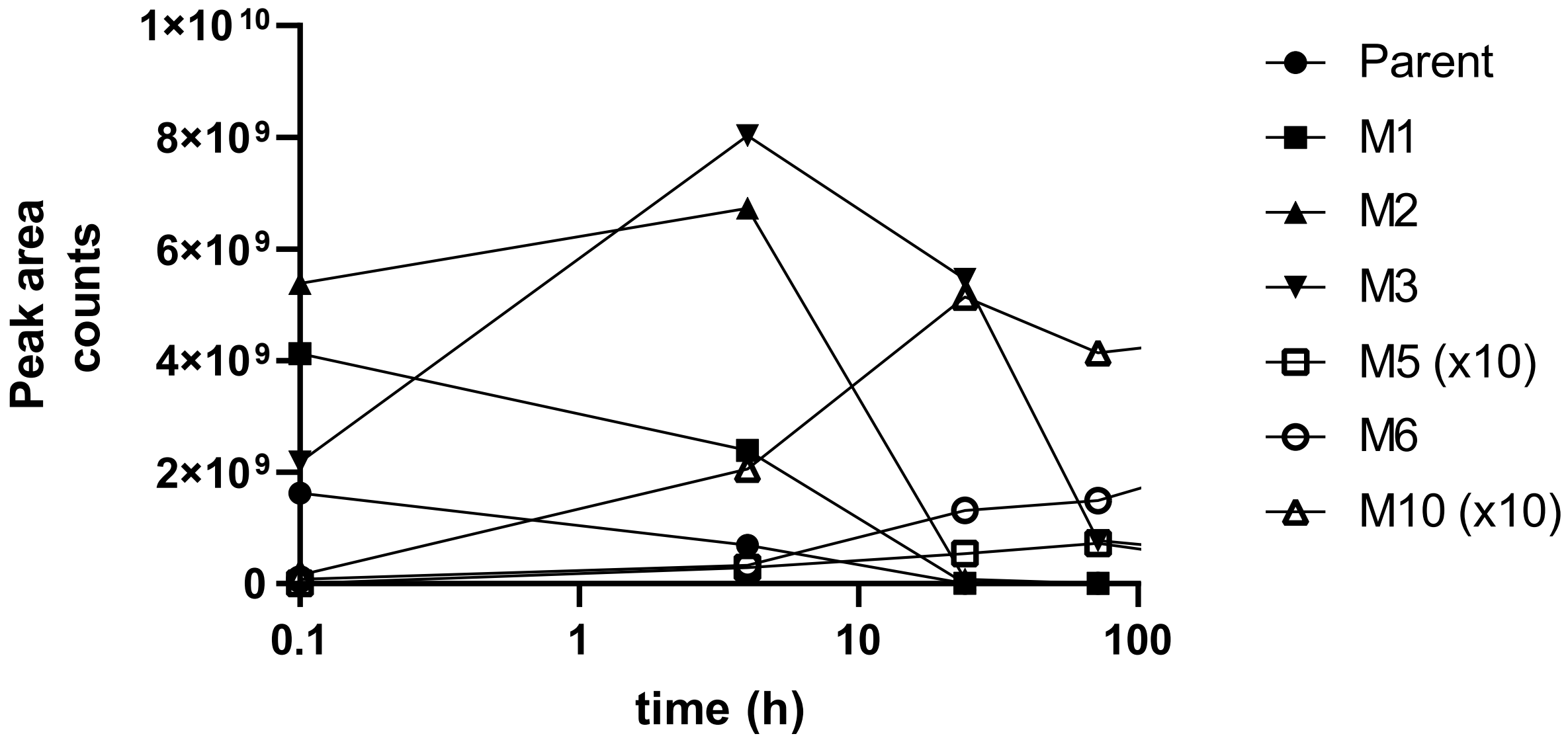

Figure S21. Time course of formation of key metabolites.
Formation of the key metabolites over time in incubations of Ambrox-5 with (-)-Ambrox. For M5 and M10 the signal is magnified by a factor of 10 for illustration. The metabolites M1 and M2 appear early and disappear after 24 h, M3 has a peak at 24 h while M5, M6 and M10 accumulate later.

Table S1. Comparison of metabolite formation by the *Sphingomonas sp.* isolates Ambrox-5 and Ambrox 29-2

		**Ambrox 29-2**	**Ambrox-5**
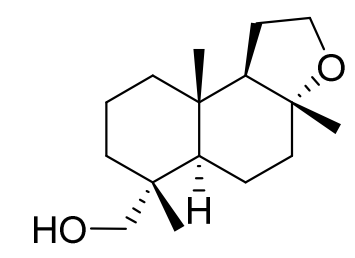	M1	4.51×10^{9} 1)	1.06×10^{10}
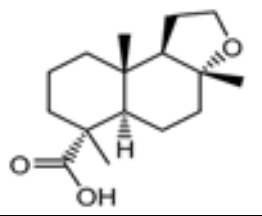	M2	1.06×10^{10}	6.63×10^{9}
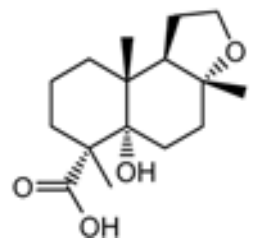	M3	1.01×10^{10}	3.62×10^{10}
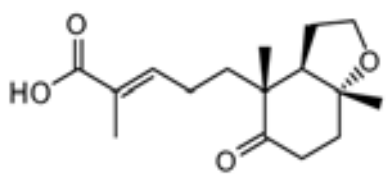	M4	2.71×10^{8}	5.28×10^{9}
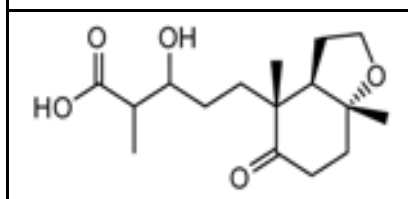	M5	1.50×10^{8}	1.73×10^{9}
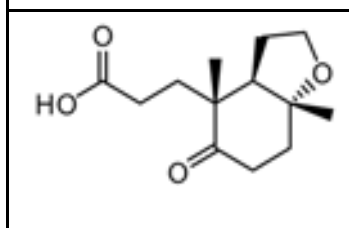	M6	1.80×10^{9}	1.77×10^{9}
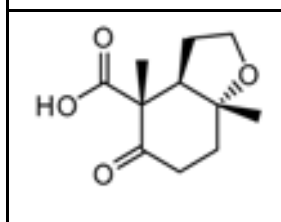	M9	b.d.	3.50×10^{6}
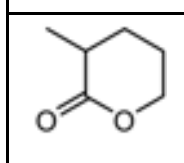	M11	2.05×10^{8}	1.59×10^{9}

1) peak area in HPLC-HRMS

Table S2. Comparison of metabolite formation by strain Ambrox-5 for the natural substrates (-)-Ambrox, Sclareol and Manool

Ambrofix			Sclareol			Manool		
Metabolite	Structure	**Peak area @ 4h**	Metabolite	Structure	**Peak area @ 4h**	Metabolite	Structure	**Peak area @ 4h**
**Parent**	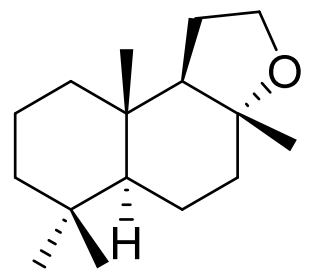	2.39E+09	**M1 [pos]**	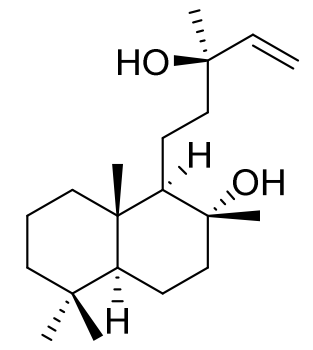	NF	**M1 [pos]**	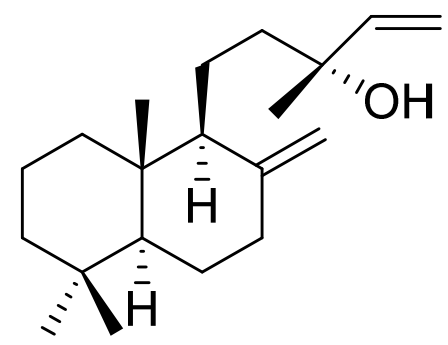	2.07E+08
**M1 [pos][1)]**	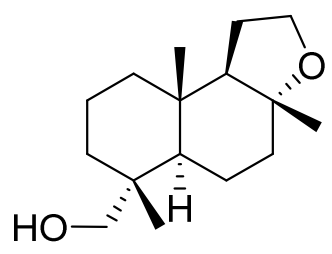	2.39E+09	**M1 [pos]**	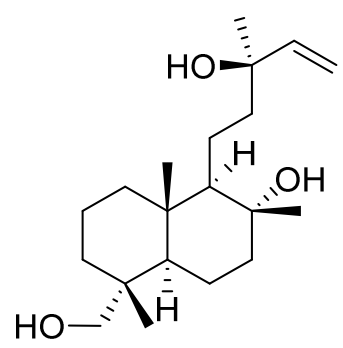	NF	**M1 [pos]**	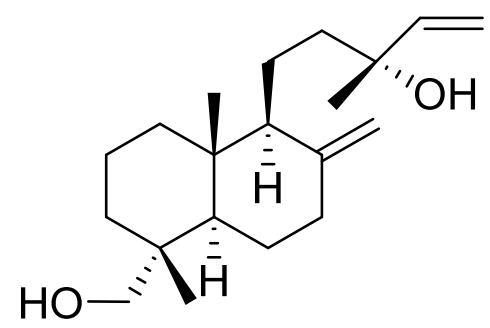	2.07E+08
**M2 [pos]**	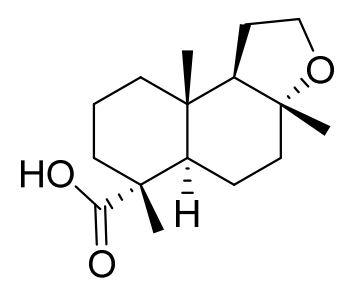	6.73E+09	**M2 [pos]**	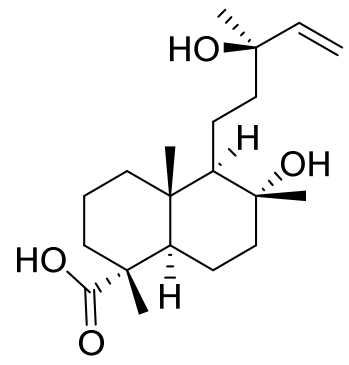	1.04E+07	**M2 [pos]**	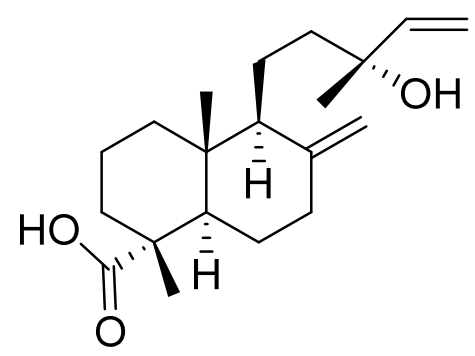	2.71E+08
			**M2 [neg]**		3.73E+08	**M2 [neg]**		8.28E+06
**M3 [pos]**	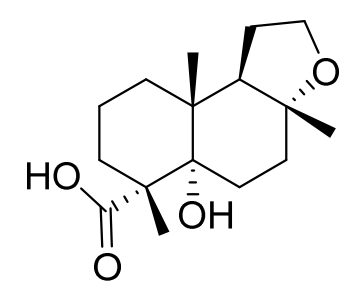	8.03E+09	**M3 [neg]**	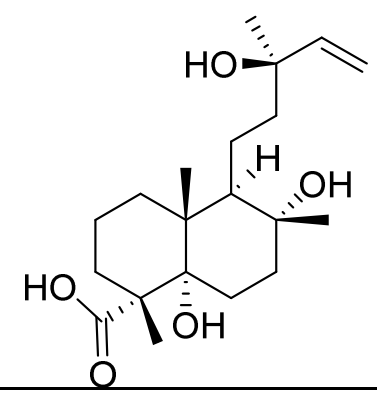	1.02E+10	**M3 [pos]**	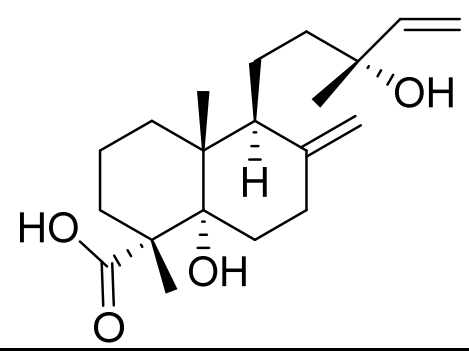	1.06E+08
						**M3 [neg]**		2.18E+09
**M4 [pos]**	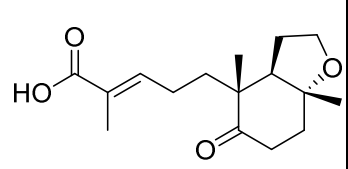	1.28E+09	**M4 [neg]**	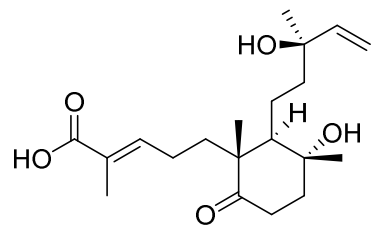	8.47E+08	**M4 [neg]**	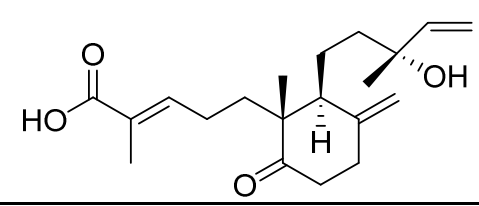	8.80E+09
**M4 [neg]**		5.75E+08	**M4 [pos]**		1.84E+07	**M4 [pos]**		2.75E+09
**M5 [pos]**	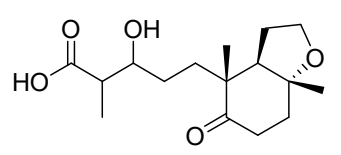	2.90E+07	**M5 [neg]**	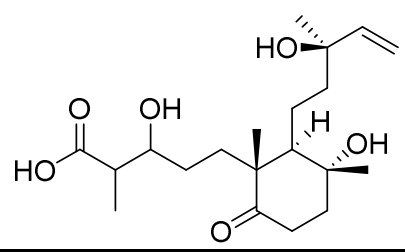	1.08E+08	**M5 [pos]**	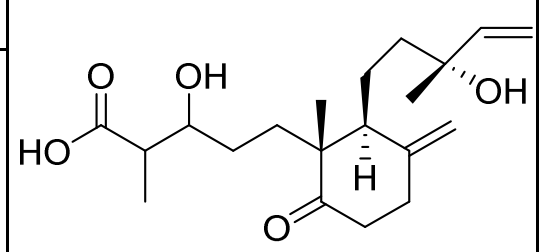	6.71E+07
**M5 [neg]**		6.66E+08				**M5 [neg]**		8.16E+08
**M6 [neg]**	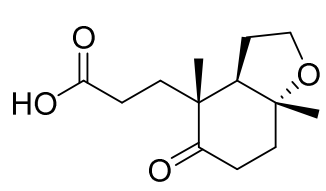	3.33E+08	**M6 [neg]**	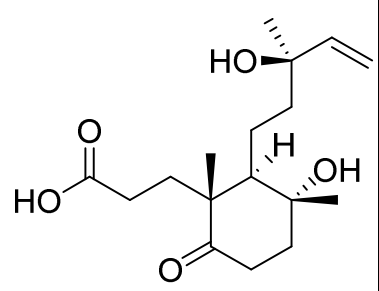	1.21E+07	**M6 [pos-RT1]**	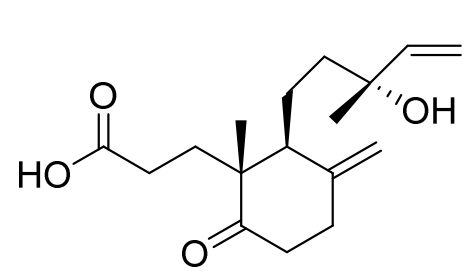	1.03E+07
						**M6 [pos-RT2]**		4.22E+07
**M9 [neg]**	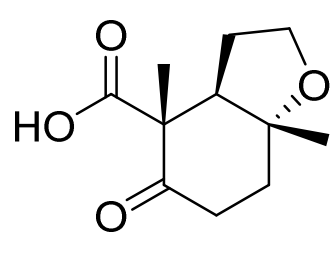	4.73E+07	**M6 [M-2H2O+H]+**	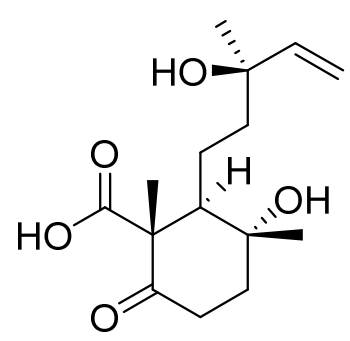	3.28E+07	**M9 [pos]**	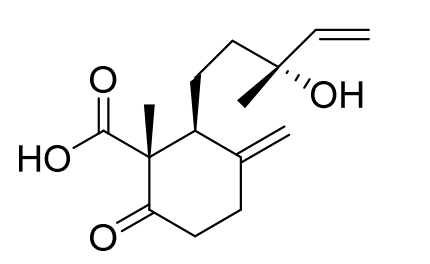	5.00E+06

[1)] Some metabolites were observed in positive and/or negative mode, and for some substrates the corresponding metabolite was observed in a different mode

4. Genetic analysis of early steps of the biosynthesis pathway

Table S3. Annotation results and assembly metrics for strains Ambrox 29-2 and Ambrox 5

Metric	Ambrox 29-2	Ambrox 5
Contigs	3	5
Total Bases	3,616,569 bp	4,829,753 bp
CDS (Coding Sequences)	3,463	4,515
tRNAs	49	50
rRNAs	6	6
tmRNAs	1	1

Table S4. Isolation of fosmid clones for the transformation of M2 to M3

		**Substrate, no bacteria T0**	**Substrate, no bacteria, T = 4 d**	**Plate 2, pool row 2 T = 4d**	**Clone [Plate 2-H2] T = 4d**	**Plate 3, pool row 12 T = 4d**	**Clone [Plate 3-C12] T = 4d**
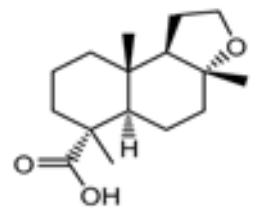	M2	5.62×10^{6} 1)	5.06×10^{6}	4.31×10^{6}	NF	3.73×10^{6}	NF
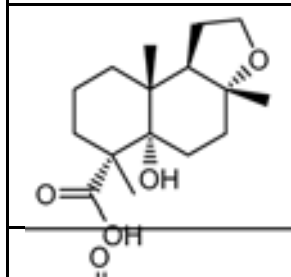	M3	NF	NF	1.86×10^{6}	5.99×10^{6}	3.13×10^{6}	8.00×10^{6}
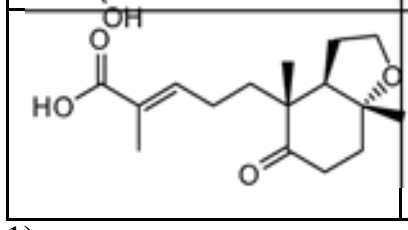	M4	NF	NF	NF	NF	NF	NF

1) Shown is the peak area in HPLC-HRMS analysis for the substrate M2, which was prepared by preparative LC from the biotransformation of (-)-Ambrox and the peak areas of the subsequent metabolites M3 and M4. Two fosmid pools and a single clone of each pool ([Plate 3-C12] and [Plate 2-H2]) after deconvolution did transform M2 to M3 but did not further transform it to M4.

Sequence information for CamC-1 and CamC-2 performing the first metabolic step to form M1:

JDHFLCAE_00007 Camphor 5-monooxygenase - camC_1

ATGATGGCCGCCCCCAACAGCTATATGCGCAGCGCCGAAGTACCGGACCACGTGCCGTCCGCGCTCGTGC

GCGACTGCAATTTCGCTCTGCTGGAAGGCGGGCAGACCGACGCGGTGGGCGCGGCGGCCCGCTACATGCG

CGACGTGCCCGACATCTTCTTCGGCGTCGGCGCGCGCCATGGCGGCAGCGCCTGGGTCGTGACGCGCAAT

GACCTGATGCGGGAAATCTATGCCGATGGCATAACCTTTTCGACCAAGGCGACTGCGAATTTTTCGGCGC

TGCTCGGCGAAAGCTGGGACTTGCTGCCGCTGGAGGTCGATCCCCCGGAAAACGCCAAGTGGCGCGACCT

TTTGAGCCCGCTCTTCTCGCCAACCCGCATGGTATCGCTGGAAAACGACATTCGTGCGACGGCGGTCGAC

ATGATCGAAGGCGTGAAAGGGAAGGGCAGGGCGGACTTCGTCTCCACCTTCTCCGCCGCTTTTCCGGTGA

CTGTTTTCCTGCGCCTGCTGGGCCTGCCGCTCGATCGGACGGGGCAGTTTCTAGACTGGGTGAACAGCCT

GCTGCACGATCCTGGAATCGAGAATAAGGTCGCCGCCGCGCATGCCATACGCTCCTACCTGGTCGAGCAG

ATGGCGATTTGCCGCGCCGCGCCGCGCGACGATGTCATCAGCAAGGTCGTGAACGGCGTCGTGGACGGGC

GTCCGGTGACGGAACAGGAATCGCTCGCCGTCTGCTTCCTTCTGTTTGTCGCGGGGCTGGATACCGTGAC

GGCGGCCCTTGGCTTTATGTTCAAGCATCTGGCCGAGCATCCCGAGCATCAGCAACAACTGCGTGAAACG
CCGTCGCTCATACCCAAGGCGGTCGAGGAGATGATGCGCGCCTATCCGGTCGTCATCAGCGGCCGGGTGC
TGACGAAGGATATCGATTTCCACGGCGTCGCGCTAAAAAAGGGCGACTCGATCATGGCGCCGACCATGTT
CGCTGGGCGGGACGAGACGAAATTCCCCCATGCCGACACCGTCGACCTGTCGCGCACGTCGATCACGCAT
CTCAGCTTCGCTGCCGGTCCGCACCGCTGCCTTGGGATGCACCTTGCCCGGCAGGAATTGAGGATCGCCA
TAGAGGAATGGCTCGCGCGCGTTCCGACCTTCAGCATTGATGCGCCGGGTCAAGCGCGCACCTACACCAC
CGGATTGTTCGGCGTCGATCACCTGCCGCTCGTATGGGCGAAAAACTGA

Molecule: **CamC-1,412 aas Protein**

Description: **Translation of CamC-1 starting at bp 1**

1 mmaapnsymr saevpdhvps alvrdcnfal leggqtdavg aaarymrdvp diffgvgarh ggsawvvtrn
71 dlmreiyadg itfstkatan fsallgeswd llplevdppe nakwrdllsp lfsptrmvsl endiratavd
141 miegvkgkgr adfvstfsaa fpvtvflrll glpldrtgqf ldwvnsllhd pgienkvaaa hairsylveq
211 maicraaprd dviskvvngv vdgrpvteqe slavcfllfv agldtvtaal gfmfkhlaeh pehqqqlret
281 pslipkavee mmraypvvis grvltkdidf hgvalkkgds imaptmfagr detkfphadt vdlsrtsith
351 lsfaagphrc lgmhlarqel riaieewlar vptfsidapg qartyttglf gvdhlplvwa kn

>JDHFLCAE_00017 Camphor 5-monooxygenase – camC-2

ATGGCTACCCAGGCTCTTCTCGATGTGCCCATGCCCACGCCCGAACCGGTCGTACTGGACGTAGACGTCC
CGGCACATGTTCCCAAACATCTCGTGCGAGATGTACGCGTGGCGCTGGGCAATGTGCCCAACACGCTCAA
CGAGCCCTATGACAATACAAAGCGGCTGCTGGAGAACGACGTCCCGCCGTTGATGTATACGCCATTTCCG
CAGGCCCAGTTTCAACAGGGGTCCTGGATCCTCACCCGCTACGCCGACGTACTGAAGGTCTATACCGACG
CGGAACTCTTTTCGACCCAGGGCGTCGCGGCGTTCCAGATCCTGGTCGGCGAGACCTGGCCCTCGCTGCC
GCTGGGGGTCGATCCGCCGCTGCACGGGCTCTACCGCCGCTTCCTCAATCCTCATTTCACGGCCAAGGCG
GTCGCGGAAATGTCCCGCGACATCCGTCAGATGTGCGTGGACATGATCGCGGAATTTCAAAAGAATGAGG
GCGGCGATTTCGCGTGGGATTTCGCGCGTGTGTTTCCGGTCCGGGTATTCTTCAACCTGATGGGCTTTCC
CATGACGGTGTTCGAGCAGTTCCTTGAGTGGGAATATCAGATCCTCCACTCGCGCGATCTCGACAAGATG
GCGGACGCGGTGCGCGGGACGATCGGCTACCTGCGTGAGTTCATCGCGGAGAAGGAGGCCAAGCCCGACG
ACACGCTGTCGAGCAAGATCGTCAACGGGACGCTCGACGGCCGTGCACTCACGAAGGATGAGCGGATCGG
CATCATGTTCTTCCTCTGGCTGGGCGGACTTGATACCGTTGCATCCACCCTGTCGCAGATGTTCCGCCGC
CTGGCGCTCGACCACGGCTTGCAACAGCGACTGCGCGACAATCCCGCGCTGATCCCGAACGCGGTCGAAG
AATTTTTGCGGATGCAGCCGCTGGTCAATTCCACCCGCTTCCTCAAAAAGGATGTGGAACTGCACGGCGT
TACGATGAAAGCGGGCGATCTGGTCTGCTGCCTGACCAGCGTCGCCAGCTTCGACCCGCTCGCCTTCCCC
GATCCACGCAACTTCGATCCTGAACGCAAGGCGATCCGCCATTTCACCTTCGCCAGCGGACCGCATCTGT
GTCTGGGCGCCCATCTGGCCCGGCAGGAGTTGAAGATCGCCCTGGAAGAATGGCTTTCGCGCGTCCCGAT
GTTCTCGATTGCAGAGGGTACGGATATGGCGGTCGTGCCTGGCCTGCTGTCGGTGCGTAACCTTCCTCTG
GTATGGGCGCAAAGTTGA

Molecule: **CamC-2,425 aas Protein**

Description: **Translation of CamC-2 starting at bp 1**

1 matqalldvp mptpepvvld vdvpahvpkh lvrdvrvalg nvpntlnepy dntkrllend vpplmytpfp
71 qaqfqqgswi ltryadvlkv ytdaelfstq gvaafqilvg etwpslplgv dpplhglyrr flnphftaka
141 vaemsrdirq mcvdmiaefq kneggdfawd farvfpvrvf fnlmgfpmtv feqfleweyq ilhsrdldkm
211 adavrgtigy lrefiaekea kpddtlsski vngtldgral tkderigimf flwlggldtv astlsqmfrr

```
281  laldhglqqr lrdnpalipn aveeflrmqp lvnstrflkk dvelhgvtmk agdlvcclts vasfdplafp
351  dprnfdperk airhftfasg phlclgahla rqelkialee wlsrvpmfsi aegtdmavvp gllsvrnlpl
421  vwaqs
```

References

Eichhorn, E., E. Locher, S. Guillemer, D. Wahler, L. Fourage and B. Schilling (2018). "Biocatalytic Process for (−)-Ambrox Production Using Squalene Hopene Cyclase." Advanced Synthesis and Catalysis **360**(12): 2339-2351.

Flachsmann, F., N. Aeberli, S. Dossenbach, L. Hortencio, H. Koch, G. Brunner, B. Spenger, E. Eichhorn, A. Fonzo, R. Berweger and D. Lelièvre (2024). "Ambrox through the Looking Glass: Chemoenzymatic Synthesis and GC-Olfactometric Analysis of 15 Ambrox Stereoisomers." Helvetica Chimica Acta **107**(7): e202400016.
